# Protein Dynamics in Individual Human Cells: Experiment and Theory

**DOI:** 10.1371/journal.pone.0004901

**Published:** 2009-04-17

**Authors:** Ariel Aharon Cohen, Tomer Kalisky, Avi Mayo, Naama Geva-Zatorsky, Tamar Danon, Irina Issaeva, Ronen Benjamine Kopito, Natalie Perzov, Ron Milo, Alex Sigal, Uri Alon

**Affiliations:** 1 Department of Molecular Cell Biology, Weizmann Institute of Science, Rehovot, Israel; 2 Department of Bioengineering, Stanford University and Howard Hughes Medical Institute, Stanford, California, United States of America; 3 Department of Materials and Interfaces, Weizmann Institute of Science, Rehovot, Israel; 4 Department of Systems Biology, Harvard Medical School, Boston, Massachusetts, United States of America; 5 Division of Biology, California Institute of Technology, Pasadena, California, United States of America; Center for Genomic Regulation, Spain

## Abstract

A current challenge in biology is to understand the dynamics of protein circuits in living human cells. Can one define and test equations for the dynamics and variability of a protein over time? Here, we address this experimentally and theoretically, by means of accurate time-resolved measurements of endogenously tagged proteins in individual human cells. As a model system, we choose three stable proteins displaying cell-cycle–dependant dynamics. We find that protein accumulation with time per cell is quadratic for proteins with long mRNA life times and approximately linear for a protein with short mRNA lifetime. Both behaviors correspond to a classical model of transcription and translation. A stochastic model, in which genes slowly switch between ON and OFF states, captures measured cell–cell variability. The data suggests, in accordance with the model, that switching to the gene ON state is exponentially distributed and that the cell–cell distribution of protein levels can be approximated by a Gamma distribution throughout the cell cycle. These results suggest that relatively simple models may describe protein dynamics in individual human cells.

## Introduction

A goal of systems biology is to understand the dynamics of protein levels, as proteins are produced and degraded. One aims for a mathematical description of these processes that captures the essentials and that can be understood intuitively.

Most current models of protein dynamics have been established and tested in micro-organisms. Proteins in bacterial cells, for example, are well described by production-degradation equations. These show that protein mass increases exponentially over time in growing cells, and that protein concentration approaches steady-state with exponential decays [1]–[5]. Such models have been shown to be useful for describing the measured dynamics of protein circuits with several interactions, such as feed-forward loop network motifs [6] and auto-regulatory loops [4], [7]–[9]. Similarly, experiment and theory has established an understanding of cell-cell variability of protein levels in bacterial and yeast cells [10]–[15]. Protein distributions in micro-organisms have been measured at steady-state and are well described by Gamma distributions [16], [17].

Much less is known about the cell-cell variability of protein dynamics in human cells [18]. Differences in expression levels between individual cells of a clonal population were previously observed [19]–[22] and related to stochastic processes. More recently, Raj *et al.* [23] have followed mRNA and protein to show bursts of mRNA production. Sigal *et al.* [24] measured variability and memory in nuclear proteins, finding that the time it takes for cells expressing a protein above or below the population mean to ‘relax’ towards the average is longer than one cell generation. More work is needed to understand the basic dynamics and differences of human protein levels and its variation across the cell cycle in individual cells.

To address this, we experimentally followed the dynamics of selected human proteins in human cancer cells and test simple models to describe these dynamics. The experiments were made possible by recent advances in obtaining dynamics of endogenously expressed proteins in individual human cells at very high resolution and accuracy [25]. This is based on a library of cell clones, each expressing a different fluorescently tagged protein expressed from its natural chromosomal location and under its native regulation. From this library, we chose three proteins that accumulate throughout the cell cycle, but are degraded every time that the cell divides. These proteins show little memory of previous cell cycles, and thus are a good starting point for testing models based on current cell properties. In the present study, we calibrated this assay to provide units of protein concentration.

We find that the proteins are found to follow either an approximately linear or a quadratic dependence on time. They also differ in their onset time of production and in their variability across the cell cycle. We find that these features can be understood in terms of a single unified model. Approximately linear accumulation results from short mRNA half-life, and quadratic accumulation from long mRNA half-life, as suggested by transcription inhibition experiments. Cell-cell variability of all three proteins is consistent with a stochastic model with slow gene ON- and OFF-transitions suggested by Raser and O'shea [14]. The measured cell-cell distribution of onset time of protein accumulation supports such slow transitions. We further show experimentally and theoretically that the protein distribution is well approximated by a Gamma distribution (described in detail at the results section) at all time points, even though dynamics are not in steady-state. Finally, we show that memory of protein levels of previous cell-cycles is lost due to the early degradation and variability in onset times of protein production. In summary, the present study presents a basis for future understanding of more complex protein circuits in human cells.

## Results

### Dynamics of PRC1, ANLN, and YB1 in individual cells

We used an approach for dynamic proteomics to measure the protein levels of PRC1 (protein regulator of cytokinesis 1, NM_003981), ANLN (anillin, NM_018685) and YB1 (Y-box binding protein-1, NM_004559) in individual living human cancer cells over several cell cycles. Cell clones were used in which these genes are tagged at their endogenous chromosomal locus with yellow fluorescent protein (EYFP) as an internal exon. The proteins are expressed as full length proteins tagged with fluorescent YFP (Figure S1). In each of the clones only one allele is tagged. The clonal cell lines in which PRC1, ANLN and YB1 are tagged originate from the same parental cell (H1299 non small cell lung cancer cell line).

The cells were followed using time-lapse fluorescence microscopy in incubated conditions controlled for temperature, humidity and CO2. This resulted in time-lapse movies over days of growth, showing about 10–20 cells per frame at a 20 minute temporal resolution. Movies were analyzed using automated image analysis software [26]. Cell segmentation was achieved by means of a second, red fluorescent labeling in these cells (see methods) that allowed automated detection of the boundaries of the cell and of its nucleus.

**Figure 1:**
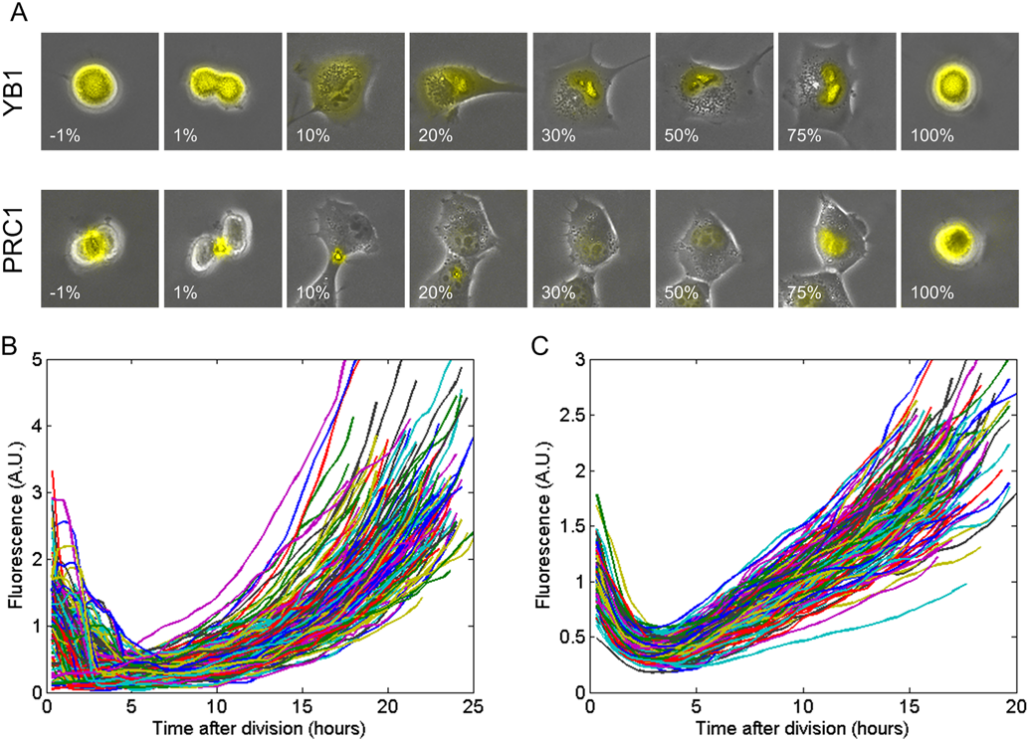
Cell cycle–dependent behavior of PRC1 and YB1. (A) Each image is an overlay of the fluorescence of the tagged protein on the corresponding phase-contrast image. One cell for each tagged protein is shown. Images are ordered according to the fraction of the time elapsed between two division events. The cells are automatically centered and neighboring cells are removed for clarity. The percentage of elapsed cell cycle is indicated at the bottom. Bar denotes 10 µm. Note the asymmetric partition of fluorescently tagged PRC1 between the two daughter cells following cell division. (B,C) For each protein, PRC1 and YB1, tracks of 150 cells are plotted. Each line denotes the total cell fluorescence level as measured during the cell cycle of a single cell. Cells are *in-silico* synchronized to beginning of their cell cycle. Each cell has different cell cycle length (PRC1: μ = 21.6±2 hr, YB1: 17.2±2 hr). Both proteins are degraded following cell division.

The three proteins showed cell-cycle dependent dynamics (Figure 1A for PRC1 and YB1 and Figure S7 and Figure S8 for ANLN). The qualitative temporal and spatial dynamics observed for all three tagged proteins agree with previous studies of the native proteins [27]–[30] and are summarized below. PRC1 functions to organize microtubules during cell division. Newly made PRC1 accumulates in the nucleus. Then, at mitosis it localizes to the spindle poles and then, during cytokinesis, it localizes to the cell midbody. Following cell division, the protein concentrated at this spot is shuttled to one or both daughter cells (for example, see Movies S1, S2, S3). Finally, the protein is degraded in the first hours following cell division [27] (Figure 1B and Figure S2A and S2C).

The second protein studied here, YB1, is a transcription and translational regulator related to cell cycle events and other processes [31]. It also shows cell-cycle dependent dynamics [32]. YB1 accumulates in the nucleus, and has a relatively constant level in the cytoplasm (Figure S2B and S2D, Movies S4, S5, S6). Like PRC1, it is degraded in the first hours following cell division (Figure 1C).

The third protein ANLN, is an actin bundling protein crucial for abscission at the final stage of the cytokinesis and is involved in coordinating the contractile activity of myosin [29], [30]. It too is degraded after cell division but accumulation over the cell cycle occurs both in the nucleus and in the cytoplasm (Figure S7A, S8A and S8B, Movies S7, S8, S9). For the sake of clarity we will focus from here on on two proteins PRC1 and YB1. Data for ANLN, whose behavior is similar to PRC1, appear in Figure S7 and Figure S8.

The degradation of these proteins in the early phase of the cell cycle is useful for the present study because it minimizes the effects of proteins carried over from previous generations. In practice, for PRC1 there was no correlation across cell cycles (R = 0.07±0.09 where R is the average Pearson correlation coefficient of accumulated protein levels at equal time points at two consecutive cell cycles of individual cells ), while YB1 showed weak correlation between daughter and mother cells (R = 0.38±0.18). See also Figure S3A and S3B for correlations across cell cycles of total protein levels and Text S1, part 1, for correlations of other parameters across the cell cycle.

### Calibration of fluorescence units in terms of protein levels

We calibrated the YFP fluorescence from the movies in terms of number of tagged protein concentrations and total number per cell. Calibration of concentrations was achieved using fluorescence correlation spectroscopy inside the cell's cytoplasm [33] (detailed in Methods). To estimate total protein in a cell, we used a nominal cell volume of 4×10^3^ µm^3^ (estimated based on the measured area of a cell ranging between 300–1500 µ^2^ and an estimated height ranging between 2–8 µ). We find that PRC1 reaches estimated maximal levels of about 250,000 molecules per cell on average, and YB1 reaches levels of 500,000 molecules per cell. Quantitatively similar calibration results were also obtained by comparing fluorescence of cells to that of purified GFP (see Methods). The calibration factor (proteins per fluorescence units) was within a factor of two for the different tagged proteins in this study.

### Average protein accumulation increases with time linearly for YB1 and quadratically for PRC1

Image analysis of the time-lapse movies allowed accurate quantitation of the total protein level in each cell, defined as the total YFP fluorescence within the cell boundaries. Cells were synchronized *in silico*, by noting the time of each division event [24], [26]. Thus, cells could be aligned according to the time that has elapsed since the last cell division (Figure 1B and 1C). Relative error of the average fluorescence measurements between day-day repeats is about 10%.

We first consider the average protein accumulation over the cell population, and then, in the next sections, the variability between cells. Protein accumulation was considered for each cell as the increase of fluorescence above the basal fluorescence at the end of the degradation event that occurs after division (see methods). The protein accumulation onset was defined for each cell as the time at which protein levels reached their minimum level. Each cell was then synchronized to the protein accumulation onset time and initial fluorescence levels were subtracted.

**Figure 2:**
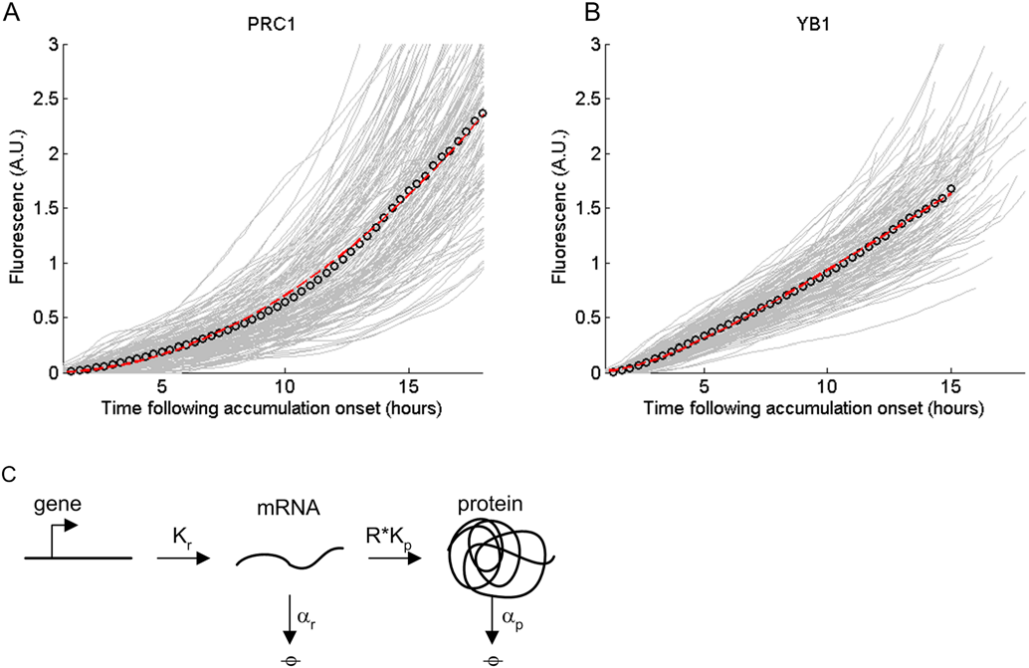
Protein accumulation profile of PRC1 is quadratic and for YB1 is linear. (A) PRC1 total cell protein accumulation exhibits an approximately quadratic profile while (B) YB1 accumulation profile is approximately linear. Each grey line denotes accumulation dynamics of an individual cell. Protein accumulation onset time was defined for all cells as the time at which protein levels reached their minimum level. Black circles denote the mean total fluorescence. Red line is the fit calculated according to a simple model of transcription and translation as schematically shown in (C).

We find that the mean protein level increases over the cell cycle. PRC1 showed a quadratic increase with time, 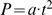, to good accuracy (R^2^ = 0.99, relative error = 2%) (Figure 2A). In contrast, YB1 showed an approximately linear increase with time, 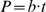, to good accuracy (R^2^ = 0.99, relative error = 1%) (Figure 2B and Figure S5).

### Model suggests that linear accumulation results from unstable mRNA and quadratic accumulation from stable mRNA

To understand the different accumulation profiles, we used a classical model of transcription and translation [34], [35] (Figure 2C). Here mRNA, r, is produced at rate k_r_ and degraded at rate α_r_, so that 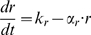. Protein is produced from the mRNA at rate 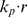 and degraded at rate α_p_, so that 
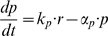
. This model makes several predictions. First, the only way to fit the non-saturating curves of both PRC1 and YB1 is to assume that protein degradation is negligible (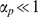). In other words, protein life-time exceeds the cell cycle, ∼20 h in the present experiment. Second, the model predicts that the quadratic accumulation of PRC1 corresponds to very long life-time of its mRNA, so that α_r_ is negligible. In this case 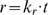, and protein increases quadratically with time 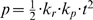.

In contrast to this quadratic accumulation, the approximately linear accumulation of YB1 is predicted to mainly result from a high degradation rate of its mRNA. In this case the mRNA concentration reaches a steady-state concentration, and as a result protein level increases linearly with time, 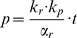.

### Experiments indicate that PRC1 mRNA is stable and YB1 mRNA is short-lived, and that both proteins are stable

To test the model predictions, we inhibited transcription by adding α-amanitin, a commonly used transcription inhibitor [36]. This drug (at 100 µg/ml) completely shuts down the action of RNA polymerase 2 in the cells, approximately 2 hours after addition [37].

**Figure 3:**
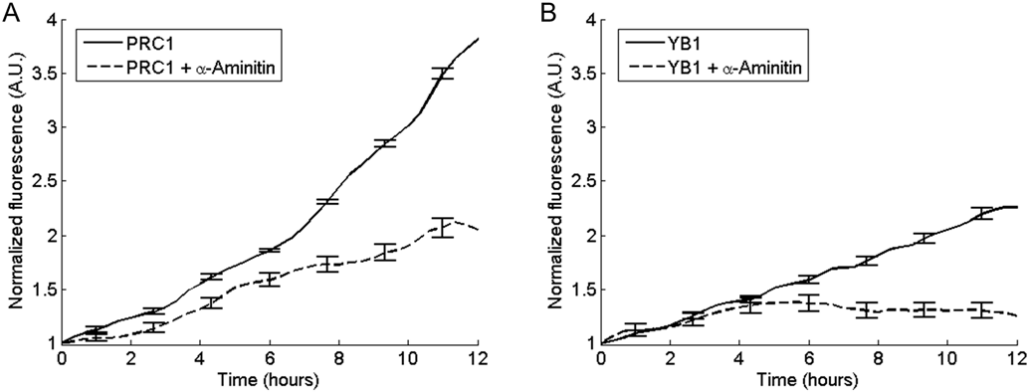
Transcription inhibitor reveals different mRNA stability for PRC1 and YB1. (A) Mean of total fluorescence of tagged PRC1 with (dashed line) and without (solid line) transcription inhibitor added at time 0 hrs. Following addition of transcription inhibitor protein levels continue to rise, though linearly instead of quadratically. This suggests that PRC1 is translated from stable mRNA. (B) Mean of total fluorescence of tagged YB1 with (dashed line) and without (solid line) transcription inhibitor added at time 0 hrs. Following addition of transcription inhibitor protein levels cease to rise, suggesting YB1 is translated from unstable mRNA. Best fit of simple differential equations regarding mRNA and protein levels suggest that the inhibitor starts taking affect at time 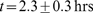 and 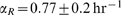.

We found that after adding α-amanitin, PRC1 protein levels began to accumulate linearly with time, as compared to the quadratic accumulation without the drug (Figure 3A). In the case of YB1, after a transient period of about 2 h after adding the drug, protein levels stopped accumulating and remained at a constant level (Figure 3B).

Fitting the differential equations describing mRNA and protein levels to measured protein levels of YB1 following addition of α-amanitin (detailed in Text S1 part 2) suggest an mRNA half life of 0.77±0.2 hr^−1^. Using this mRNA life-time in equations describing behavior in un-perturbed cells, we find that mRNA levels reach 90% of steady state in 3 hrs and 99% in 6 hrs. These results agree with the model predictions, suggesting stable mRNA for PRC1, short-lived mRNA for YB1. That both proteins are highly stable is evidenced by the fact that their levels do not decrease following inhibitor addition. In summary, this experiment supports the simple model predictions.

### Experiments suggest that PRC1 (and ANLN) mRNA degrades shortly after cell division

**Figure 4:**
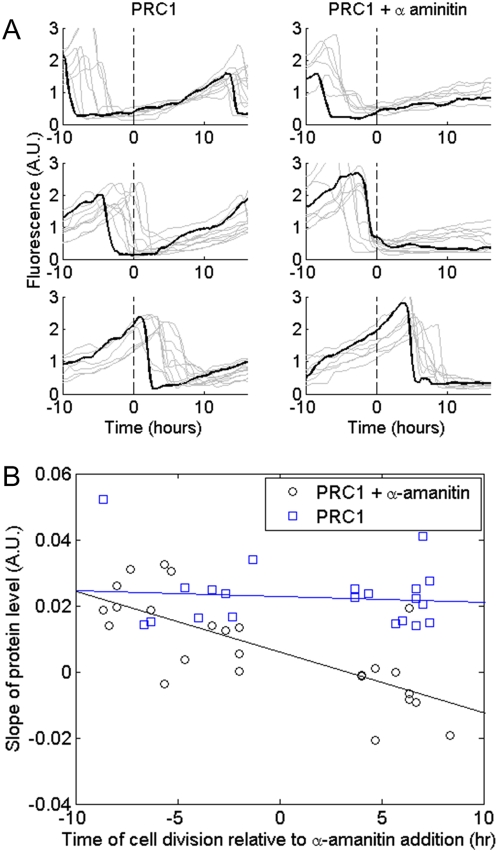
Transcription inhibitor reveals PRC1 mRNA degrades shortly after cell division. (A) Cells are classed into three groups based on the time of their division relative to the time of administration of α-amanitin, a transcription inhibitor (time = 0 hrs). Top group – division *before* inhibitor addition, middle group – division *during* inhibitor addition and bottom group – division *after* inhibitor addition. Right panel – 2 ml medium was exchanged with 2 ml fresh medium containing 100 µg/ml α-amanitin (at t = 0 hrs), Left panel – 2 ml medium was exchanged with 2 ml fresh medium (at t = 0 hrs. mock control),. Each grey line denotes measurements of a single cell, black line is a selected cell for eye guidance. (B) Cells dividing at later stages, relative to the time the transcription inhibitor was added, show reduced protein production (Pearson correlation R = −0.73 p<0.01). Circles and squares denote rates of protein production with and without a transcription inhibitor, respectively. Gray lines denote best linear fit to each of the data sets.

*In–silico* synchronization of trajectories of individual cells, following addition of the transcription inhibitor α-amanitin, allows studying effects of cell-cycle phase on the dynamics of the mRNA. For each cell, we determined the time that elapsed between its previous division and the time at which the drug was added. In the case of PRC1 and ANLN, we found that cells, for which transcription was blocked shortly after division, showed no protein accumulation. In contrast, cells in which transcription was blocked several hours after cell division showed significant accumulation of protein that rose linearly with time (Figure 4A and 4B and Figure S4 for PRC1 and Figure S8C for ANLN).

This indicates that mRNA is degraded after cell division. This agrees with previous micro array experiments on synchronized HeLa cell populations that show a sharp drop in PRC1 and ANLN mRNA levels after cell division [38]. These findings support the use of zero initial mRNA levels following cell division in the theoretical models. In the case of YB1, no protein accumulation was observed following addition of transcription inhibitor at any stage of the cell cycle suggesting short mRNA half life.

### Protein accumulation varies from cell to cell

So far we have discussed the average behavior of the cell population observed in the movies. We now turn to analyze the *variability* between different individual cells within the same clone. We find that cells show a spread of trajectories around the mean (Gray lines in Figure 2A and 2B). The individual-cell trajectories of PRC1 are typically quadratic with time to a good approximation (best fit of 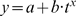 to individual cell data is for 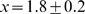). The individual-cell trajectories of YB1 have an approximately linear dependence on time (best fit of 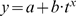 to individual cell data is for 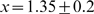) (Figure S6). Thus, each cell follows the mean behavior, but with somewhat different slope and (in the case of PRC1), curvature.

**Figure 5:**
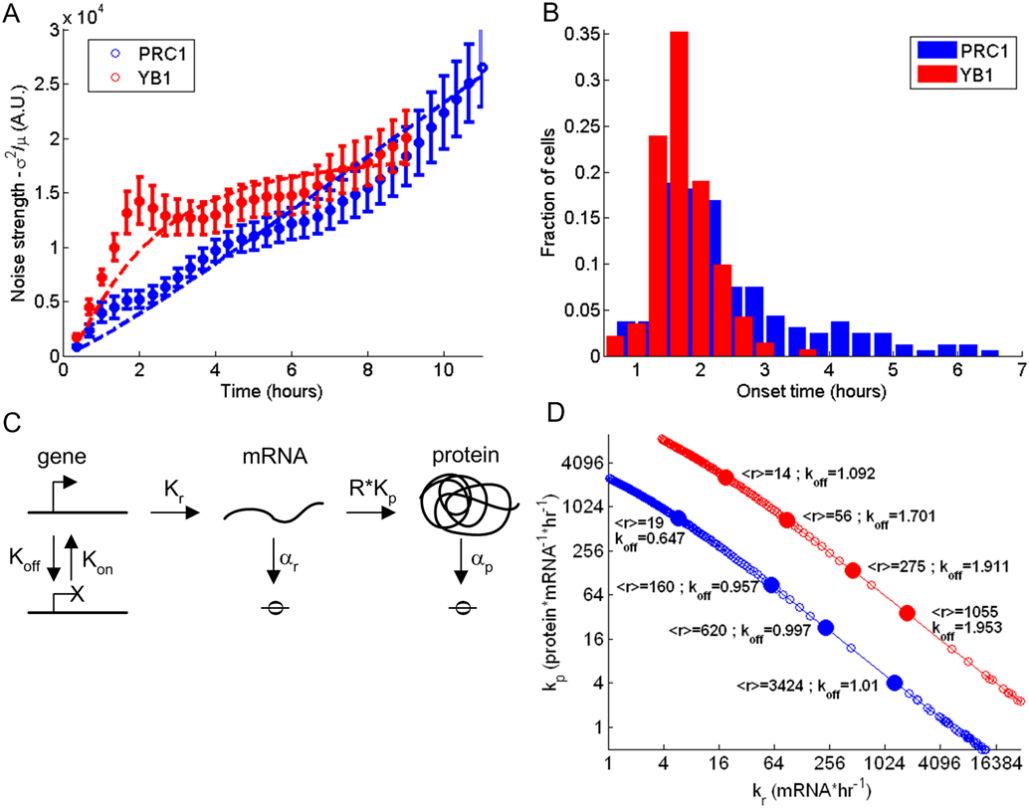
Variability of protein levels and accumulation onset times differs between PRC1 and YB1. (A) Noise strength (std^2^/mean) of PRC1 rises linearly (blue circles) and of YB1 saturates (red circles). Both 2-step and 3-step stochastic models reproduce these profiles of noise strength (blue solid line for PRC1 and red solid line for YB1). (B) Time interval between end of degradation to beginning of accumulation for PRC1 in blue and YB1 in red. The time of accumulation has an exponential tail (decay rate of 0.68±0.24 hr^−1^ for PRC1 and of 1.88±0.44 hr^−1^ for YB1). (C) Schematic of three stage model, with slow transitions between open and closed DNA. (D) Parameter estimation based on the slow switching model. As explained in the text, several parameters are estimated directly from the data, for PRC1; k_on_ = 0.68±0.24, α_r_ = 0 and α_p_ = 0. For YB1; k_on_ = 1.88±0.44, α_r_ = 0.77±0.2 and α_p_ = 0. For the rest of the parameters, the model was fit to data (mean and noise strength) and k_p_ and k_r_ were determined for a range of k_off_. Red and blue circles denote YB1 and PRC1 respectively. Values of average mRNA levels over the cell cycle and k_off_ are shown for several parameters sets (filled circles).

We quantified this variation between cells using the noise strength, defined by ratio of the variance to the mean. Thus, noise strength, is NS = variance/mean. We note that a second common measure, the coefficient of variation CV = std/mean was more susceptible to noise in the present data. We find that the NS increased over time for PRC1, and appeared to saturate with time for YB1 (Figure 5A, circles). Thus, noise has different dynamics for the two proteins.

We also found that different cells began to significantly accumulate the proteins at different times after cell division. In particular, there appeared to be a large cell-cell variation of such ‘onset times’ for PRC1 (Figure 5B). Some cells showed protein accumulation after an hour and others after five or more hours. We measured the distribution of the time at which protein levels first begin to rise in each cell. We find that this distribution has an exponential tail, with a decay time of 1.02±0.36 hrs. For YB1, we also find an exponential tail but with a shorter decay time 0.37±0.09 hrs. This indicates that timing is less variable between cells for YB1 (Figure 1C). For more details, see Text S1, part 7.

### A model based on slow gene switching captures the variability

To understand the present data on cell-cell variability, we employed models for stochasticity in protein expression. The observed exponential distribution of times for the onset of protein accumulation is consistent with a slow process that turns expression ON with a timescale of hours. This corresponds to a picture, suggested in previous studies [11], [14], [20], and observed in other studies [23], [39], in which genes switch slowly from ON to OFF states, and transcription occurs from the ON state alone (Figure 5C). We find that such a stochastic model captures the present data on noise strength (Figure 5A, solid lines), with parameter sets shown in Figure 5D.

We also compared the data to a model with no gene switching. This model is a straightforward stochastic version of the model of Figure 2C where each rate is interpreted as a probability per unit time. We found that such a model can only explain the extent of the observed variability when one uses parameters that give very low mRNA levels: only a few mRNA/cell leading to high variability due to small-number fluctuations. Given our estimates of several hundred thousand proteins per cell, this leads to a very high translation rate in the model, in order to provide the observed number of proteins/mRNA. Such a rate is at the limit of physical limitations of the cell (maximal ribosomal packing along the transcript, in Text S1 part 8).

In summary, the slow-switching model in which genes switch from OFF to ON on the timescale of hours seems to generate the large observed variability naturally, because of the long times between opening and closing events, resulting in relatively rare bursts of mRNA production [15], [17], [23], [39], [40]. Both models are detailed in Text S1 parts 3 and 4

### Cell–cell protein distributions match theoretical prediction of Gamma distributions

Theoretical studies [10], [17], [40] have noted that the stochastic model employed in our study should result in a cell-cell distribution of protein levels that follows a Gamma distribution (or, for very small protein numbers a related distribution known as the negative binomial distribution [16]). Gamma distributions are single-peaked distributions. They have a sharp peak and an exponential tail at low average protein numbers, and have a bell-shaped distribution at high average protein numbers. The formula for the probability of having n proteins has two parameters, a and b, and is:
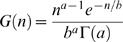
where a = m^2^/σ^2^ and b = σ^2^/m (m and σ denote mean and standard deviation respectively). These theoretical results were obtained for steady-state processes. Here, we extended this theoretical analysis also to the case of dynamics that are not in steady-state. The present system is out of steady state, because the average protein level rises with time. We used a moment analysis to determine the distribution at different time points (See Text S1 part 5). We found that after a short transient time, the predicted cell-cell distributions *at each time point* are close to a Gamma distribution, with parameters that vary over time.

**Figure 6:**
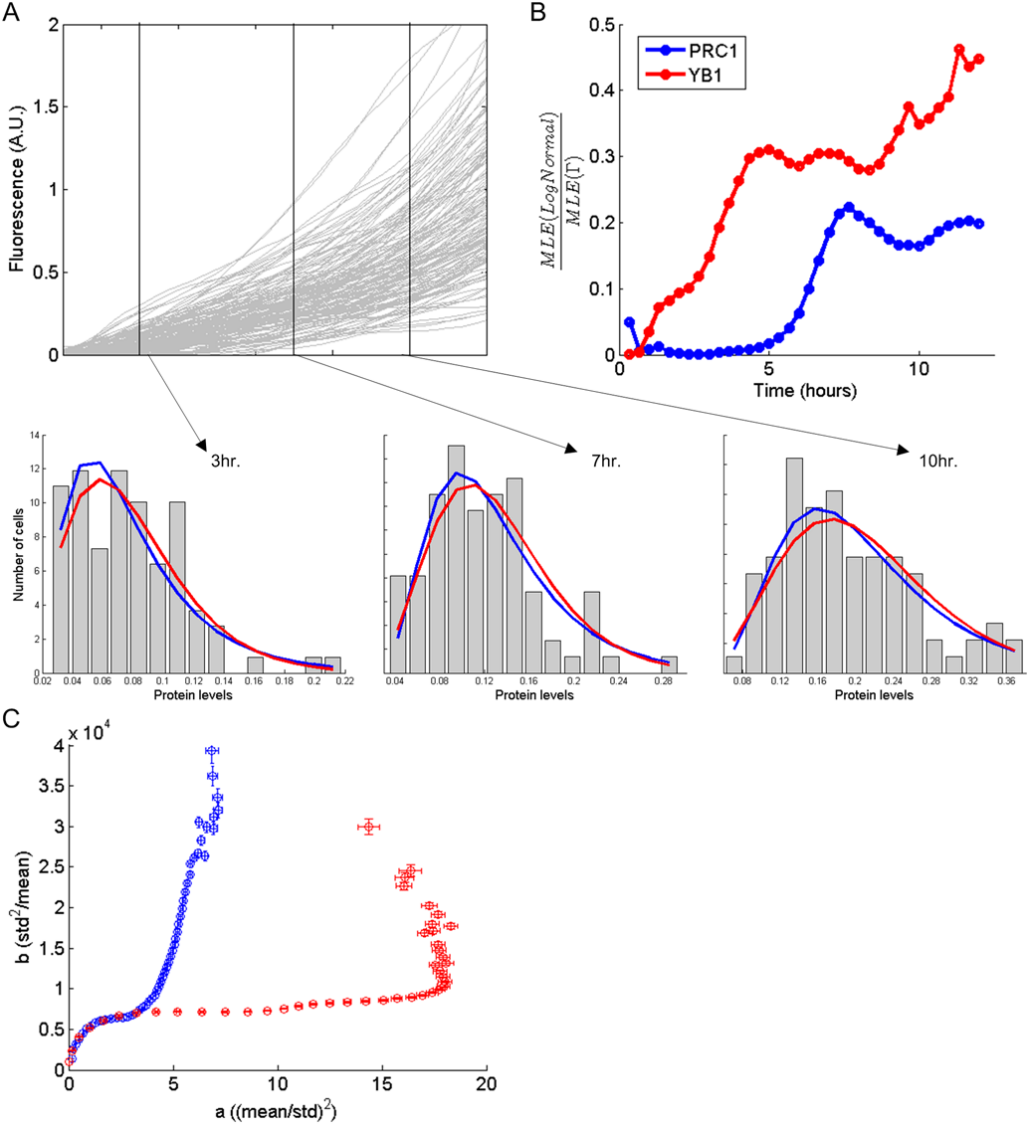
Cell–cell protein distributions match theoretical prediction of Gamma distributions with time-dependant parameters. (A) Example of cell tracks (each line denotes PRC1-tagged fluorescence measured in a single cell), Distributions of fluorescence levels at 3, 7 and 10 hours following accumulation onset are shown. Fit to log-normal (red) and Gamma (blue) distributions are overlaid. (B) Ratio between maximum likelihood estimation (MLE) of log-normal and gamma distributions was computed at a temporal resolution of 20 minutes for PRC1 (blue) and YB1 (red). Cell-cell distributions fit Gamma distribution better (ratio<1) across the cell cycle. (C) Variation of the effective Gamma distribution parameters *a* (scale), and *b* (shape). These parameters are related to the mean, μ, and standard deviation, σ, by 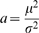 and 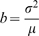. Bold blue and red circles denote *a*,*b* measurements for YB1 and PRC1 respectively for the first 12 hours of protein accumulation. Error bars denote standard errors.

We found that Gamma distributions describe the observed experimental data well at all times. Gamma distributions seem to describe the data better than other empirically suggested distributions, such as Gaussian or log-normal distributions (Figure 6A and 6B). The two parameters that define the Gamma distribution (a and b), corresponding to the mean protein level and the noise strength, vary over time (Figure 6C). We found that these parameters vary in a different way for PRC1 and YB1, changing in almost an orthogonal manner. These two parameters were interpreted by Friedman et al [17], [40] to represent burst size (average number of protein molecules per burst) and burst frequency (average frequency of expression bursts per cell cycle) respectively.

### Cellular mixing occurs at early stages of the cell cycle and is independent of protein accumulation patterns

Tracking changes of protein levels at the individual cell level over time allows following the mixing of protein level ranking between cells over time.. Cellular mixing occurs when a cell lineage, given enough time, reaches the different states found in a snapshot of a cell population. Mixing was measured using two approaches (detailed by Sigal *et al.* [24] and in methods). The first uses the auto-correlation function A(τ) of the protein levels. The second, an ergodic metric, ranked the cells according to tagged protein fluorescence at the beginning of the movie, and followed the fraction of the total ranks that each cell traversed as a function of time (see Methods).

**Figure 7:**
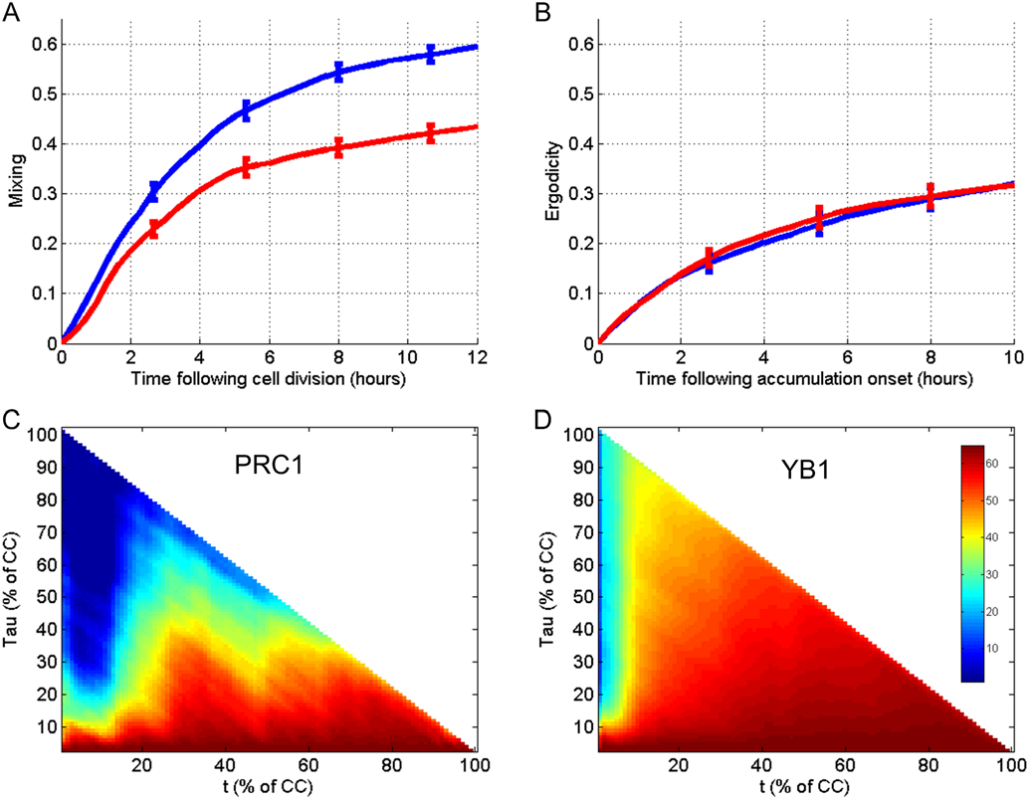
Single-cell tracks reveal different mixing patterns for PRC1 and YB1. Mixing is measured by auto-correlation and an ergodicity measure. In both measurements, cells are ranked according to tagged protein fluorescence at the beginning of the movie. Ergodicity is defined as the measure of the fraction of the total ranks that each cell traversed as a function of time. (A) Mixing of cells synchronized to the *beginning of cell cycle* is measured by the ergodicity as a function of time (for 12 hours) for PRC1 (blue) and YB1 (red). (B) Mixing of cells synchronized to *accumulation onset* is measured as a function of time (for 10 hours) for PRC1 (blue) and YB1 (red). (C, D) Cells were synchronized *‘in-silico’* by aligning the dynamics between two division events and the fraction of elapsed cell cycle was used as a time base. Auto-correlation matrix for each time point (percentage of cell cycle) and all following time points is shown for YB1 in (C) and PRC1 in (D).

We measured mixing across the entire cell cycle and during the shorter period of protein accumulation. While cells exhibit faster mixing for PRC1 at the beginning of the cell cycle compared to YB1 (Figure 7A), similar mixing was observed for both proteins in the protein accumulation phase (Figure 7B), despite their different accumulation rates (approximately constant for YB1 and linear for PRC1). Loss of cellular memory of previous protein levels seems to occur at early stages of the cell cycle due to degradation and varying onset times of accumulation. Mixing occurs to a much lesser extent in the accumulation phase of newly synthesized proteins (Figure 7C and 7D).

## Discussion

This study presented an experimental and theoretical analysis of the detailed dynamics and variability of selected proteins in human cells. This analysis was made possible by an experimental advance that allows highly accurate, time-resolved measurement of proteins expressed from their native chromosomal position, and under their natural regulation, in individual cells. We chose proteins that are degraded upon cell division and that accumulate throughout the cell cycle, and which do not significantly carry memory of their levels in the previous cell cycle.

We find either a linear accumulation with time or a quadratic accumulation with time. Linear accumulation is found to correspond to short mRNA life-time, and quadratic accumulation to long mRNA life-time, in accord with simple transcription-translation equations. The theoretical predictions were tested by using a transcription inhibitor, which showed how quadratic profiles turn to linear, and linear profiles turn to constant protein levels, as predicted.

Note that linear and quadratic accumulation of total protein/cell are quite different from the typical patterns found in bacteria, in which total protein levels typically rise exponentially (or nearly exponentially) across the cell cycle.

We also studied the cell-cell variability in the protein levels. A model with slow transitions between ON and OFF gene states captures the observed variability, including the exponential distribution of onset times and the Gamma-shaped cell-cell distribution of protein levels. The extent of the noise seems to be too large to be explained by stochastic models that lack slow ON-OFF transitions.

In addition to single-time point statistics, we also considered the rate at which protein levels mix so that cells higher or lower than average return to average. Except for a short period after cell division, in which memory of previous levels is rapidly lost, the protein levels seem to show a small extent of mixing, with accumulation trajectories that are similarly shaped and roughly parallel for different cells in the population. Thus, the proteins display distinct cell-cell individuality over the cell cycle.

One may ask whether the different accumulation profile shapes we observed for YB1 and PRC1 correspond to their biological function. PRC1 is essential for the final stage of the cell cycle [41], [42], where it acts to control the spatiotemporal formation of the midzone during cytokinesis. In contrast, YB1 is involved in processes throughout the cell cycle [31], such as transcriptional regulation, translational regulation, DNA repair, stress response and cell proliferation. A quadratic accumulation as in PRC1 prepares most of the protein in the last stage of the cell cycle. A linear accumulation profile as in YB1 may be better suited for proteins that need to serve the gradually growing cell content across the cell cycle.

This study raises the hope that relatively simple models might accurately capture the behavior of protein dynamics. Future work can explore whether such models can be used as building blocks to understand more complex protein circuitry. It would be important to further test this approach by measurements of additional proteins. For this purpose, the present experimental approach may be of use, especially in conjunction with a comprehensive library of tagged human proteins [25], to characterize the mean and individual cell behavior under diverse conditions.

## Methods

### YFP CD-tagging of endogenous proteins

PRC1 and YB1 were chosen from a library of tagged proteins in the H1299 non-small cell lung carcinoma cell line. This library was constructed as described in Sigal *et al.* [26]. Briefly, the enhanced yellow fluorescent protein coding region (EYFP), flanked with splice acceptor and donor sequences, was placed on a retroviral vector (pBabeAE) and integrated into the genome. Three vectors were used, one with EYFP in each reading frame. EYFP is expressed when the virus integrates into an intron of an expressed gene in the right frame and orientation, leading to incorporation of YFP as a new exon in the spliced mRNA. This method is known as Central-Dogma tagging (CD-tagging) [24]–[26], [43], [44]. Cells positive for YFP fluorescence were sorted using flow cytometry into 384-well plates and grown into cell clones. Tagged protein identities were determined by 3′-RACE (rapid amplification of cDNA ends), using a nested polymerase chain reaction (PCR) that amplified the section between EYFP and the polyA tail of the mRNA of the host gene. PCR products were sequenced directly and aligned to the genome.

### Image analysis using a red tag in the parental cell line

The present library has an additional feature which is crucial for accurate image analysis, and which goes beyond [26] : the parental cell was tagged with a second red fluorescent tag, mCherry. This tag was inserted by two rounds of CD tagging into two genes, one expressed in the nucleus and the other expressed in the entire intracellular volume. This tagging was used in the parental cell of the present library in order to enable computerized segmentation of the whole cell. Our previous studies used the EYFP tag for image analysis [24], [26] and cell tracking, but was generally limited to cells with nuclear protein localization. The present red tag allows automated analysis of cells with arbitrary EFYP protein localization. There was a need for tagging two proteins with mCherry for segmentation purposes; the cytoplasmic red tagged protein enabled segmentation of cells from background, while the stronger nuclear tag served both as a seed for each cell in the segmentation process and enabled segmentation of nucleus from cytoplasm. The library of CD-tagged proteins with a red tag currently includes over 1300 unique proteins and is detailed at http://www.dynamicproteomics.net.

### Time-lapse microscopy

Time-lapse movies at 20× magnification were obtained as described [26] with an automated, incubated Leica DMIRE2 inverted fluorescence microscope and an ORCA ER cooled CCD camera (Hamamatsu Photonics). The system was controlled by ImagePro5 Plus (Media Cybernetics) software which integrated time-lapse acquisition, stage movement, and software-based auto-focus. During the experiment, cells were grown and visualized in 12-well coverslip bottom plates (MatTek) coated with fibronectin (Sigma). The standard RPMI medium was replaced with RPMI medium without phenol red (Biological Industries) to decrease autofluorescence. For each well, time lapse movies were obtained at four fields of view. Each movie was taken at a time resolution of 20 minutes and was filmed for at least three days (over 200 time points). Each time point included three images- phase contrast, red and yellow fluorescence.

### Transcription inhibition

α-amanitin (A2263 Sigma), was dissolved in DMSO (hybri-max, D2650 Sigma) for a stock solution of 1 mg/ml. Cells were grown and filmed as explained in previous sections. After ∼24 hours of growth in the microscope, growth medium (2 ml) was gently replaced by growth medium with α-amanitin (100 µg/ml in 2 ml) under the microscope.

### Image analysis of time-lapse movies

Custom image analysis software performed cell tracking and segmentation, and background and bleaching corrections (only very low levels of bleaching ∼3% occur in this experiment). Segmentation was applied to images after flat-field correction and background subtraction [26]. A seeded watershed segmentation algorithm [45]–[47] was carried out on the red fluorescent images using the strong fluorescence of the nuclei as seeds. Nuclei were seperated from cytoplasm using the sharp difference of fluorescence between the nucleus and cytoplasm. Tracking of cells was performed by analysing the movie from end to start and linking each segmented cell to the cell in the previous image with the closest centroid. Cell divisions were automatically detected by a sharp twofold drop in total fluorescence between consecutive images [26]. Tracking, bleaching and image corrections are described in detail in [26]

### Protein accumulation onset times

Protein accumulation onset was defined for each of the cells as the time at which protein level reached its minimum level. To compute the time delay between end of degradation to beginning of accumulation, the minimum level of each cell was subtracted from its profile. A threshold chosen to be 1 percent of the maximum mean protein levels was computed. For each cell the first and last time points in which the threshold was crossed were denoted as the end of degradation and beginning of accumulation phases, respectively. Results are robust to threshold levels between 0.03–3% of the maximum level.

### Calibration of YFP fluorescence to protein molecules

Calibration between number of EYFP molecules and measured fluorescence levels was carried out using Fluorescence Correlation Spectroscopy (FCS) [48] for the protein YB1. The FCS specific setup is detailed at Kopito *et al.* [33]. Cells were grown on standard 1-×3-inch microscope slides and prior to measurement were covered by glass coverslips and sealed immediately with paraffin wax. Then laser illumination was calibrated to photon counts per YFP in the YB1 sample.(about 400 photons per molecule within illumination volume). Once calibrated, measurements were taken from the cytoplasm of ∼20 cells (cytoplasm concentrations are relatively constant throughout the cell cycle). Mean photon count in laser illumination volume (0.25 µm3) is 15,000 and equivalent to about 37 protein molecules. Using mean cell volume to be 20×20×10 µm and cytoplasm to constitute 2/3 of the cell volume and that protein amount in the cytoplasm is 1/5 of total protein level at end of cell cycle (see Figure S2), sums up to ∼500,000 molecules in cell at end of cell cycle. Mean total fluorescence at end of cell cycle is ∼400,000 thus leading to ∼0.8 fluorescence counts per molecule.

A second method for calibration used purified protein standards in the following way: 5 µL of purified GFP solution containing ∼10^12^ molecules of GFP were placed under a slide cover slip with diameter of 13 mm. Images with linearly increasing exposure times were taken in order to compute the gain of the mean pixel level. Number of GFP molecules per pixel was computed (∼7700) and compared to the mean pixel fluorescence (∼3000 fluorescence A.U. per 1000 msec exposure), and summed up to ∼0.4 fluorescence counts per molecule.

### Estimation of of mRNA life time after cell division

Time-lapse movies were acquired for several hours in 2 ml RPMI medium. Then the medium was switched to 2 ml of medium containing 100 nM of α-amanitin and time –lapse movie acquisition was continued. Cells were manually chosen so that only cells that were in the accumulation stage and did not divide for 8 hours following drug adittion were used. To compare to theory, mean of data was fit to two functions. The first describing the mean behavior until the inhibitor becomes fully active (∼2 hrs) and the second from that stage allowing mRNA degradation rate to be obtained (see Text S1, part 2).

### Auto-correlation calculation

The auto-correlation function was computed with a robust estimator 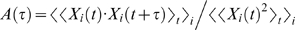, where averages over time and over cells are denoted <>_t_ and <>_i_ respectively. We measured the auto-correlation of the ranked total fluorescence *R_i_(t)*, such that 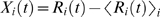. This method was less sensitive to outliers in the data than auto-correlation over the total fluorescence [24], [49].

### Mixing (ergodic metric) calculation

The ergodic metric 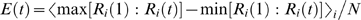 where the ranked total fluorescence at time t is denoted *R_i_(t)*, average over cells is denoted <>i and N is the number of cells [24]. Thus this metric ranks the cells according to tagged protein fluorescence at the beginning of the movie, and follows the fraction of the total ranks that each cell traverses as a function of time.

## Supporting Information

Figure S1 Immunoblots of YB1, PRC1, and ANLN with anti-GFP. Estimated molecular weight of each protein without yellow fluorescent tag is: YB1 ∼36 kDa, PRC1 ∼71 kDa and ANLN ∼124 kDa. YFP ∼27 kDa.
(1.85 MB TIF)

Figure S2 Protein distribution within cellular compartments during cell cycle. (A,B) Mean of total fluorescent levels in different compartments of the cell (cyan - cytoplasm, magenta - nucleus and black- whole cell) for PRC1 and YB1. (C,D) Mean of mean fluorescent levels in different compartments of the cell (cyan - cytoplasm, magenta - nucleus) for PRC1 and YB1. (E) Mean of total fluorescent levels of 2 proteins tagged with mCherry in the PRC1 and YB1 clones. The mCherry fluorescence and distribution is used for cell segmentation and tracking.
(1.85 MB TIF)

Figure S3 Correlation across consecutive cell cycles. (A,B) cells were tracked for two consecutive cell cycles. Shown is the correlation matrix of each time point in first cell cycle and all time points in the second cell cycle for PRC1 and YB1 respectively. Note that PRC1 doesn't retain any memory across cell cycles while YB1 shows relatively high correlation between fluorescent levels of a mother cell and fluorescent levels in daughter cell.
(1.73 MB TIF)

Figure S4 Response of single cells expressing tagged PRC1 at different stages of their cell cycle to transcription inhibitor reveal mRNA degradation at beginning of cell cycle. (A) Cells were divided into three groups based on the time of their division relative to the time of administration of α-aminitin, a transcription inhibitor (time = 0 hrs). Top group - division before drug addition, middle group - division during drug addition and bottom group - division after drug addition. Left panel - 2 ml medium was exchanged with 2 ml fresh medium (at t = 0 hrs), right panel - 2 ml medium was exchanged with 2 ml fresh medium containing 100 µg/ml α-aminitin (at t = 0 hrs). Each grey line denotes measurements of a single cell, black line is a chosen cell for eye guidance. Note that as cell divide later in the experiment the slope of protein levels decreases. (B) all trajectories of right panel overlaid and color ordered based on time of cell division, early divisions in blue and late divisions in red.
(2.21 MB TIF)

Figure S5 Single cell trajectories display linear profiles in the case of YB1 and quadratic profiles in the case of PRC1. Each single cell trajectory denoting fluorescent levels measured over the cell cycle was fit to a polynomial function of first and second degree. Plotted are the pearson correlation coefficient values (R^2^) of the experimental data and the fit for PRC1 (red) and YB1 (blue). All trajectories of YB1 showed R^2^>0.9 already in the linear fit, while PRC1 showed R^2^>0.9 for all cells only when using a polynomial of 2^nd^ degree.
(0.71 MB TIF)

Figure S6 Coefficient of Variance (CV) of PRC1 and YB1. CV of PRC1 (blue) and YB1 (red) of protein levls across cells synchronized to the beginning of protein accumulation. Inset denotes the CV of the first 4 hours.
(1.35 MB TIF)

Figure S7 Cell cycle dependent behavior of ANLN. (A) Example of one cell automatically tracked through the cell cycle. Each image is an overlay of fluorescent tagged protein on phase contrast image. Images are ordered according to the fraction of the time elapsed between two division events. The cells are automatically centered. The percentage of elapsed cell cycle is indicated at the bottom, Bar, 10 µm. Note asymmetric division of fluorescently tagged PRC1 between the two daughter cells following cell division. (B,C) For each protein, PRC1 and YB1 tracks of 150 cells are plotted. Each line denotes the total fluorescence level as measured during the cell cycle of a single cell. Cells are synchronized to beginning of cell cycle. Each cell has different cell cycle length (PRC1: μ = 21.6±2 hrs, YB1: 17.2±2 hrs). Both proteins are degraded following cell division.
(1.52 MB TIF)

Figure S8 Cell cycle dependent behavior of ANLN. (A) Mean of total fluorescent levels in different compartments of the cell (cyan - cytoplasm, magenta - nucleus and black- whole cell). (B) Mean of mean fluorescent levels in different compartments of the cell (cyan - cytoplasm, magenta - nucleus). (C) The same as Legend of figure S5A just for ANLN.
(0.10 MB TIF)

Text S1 (0.61 MB PDF)

Movie S1 Time-lapse movie of transmitted light images of the clone with YFP CD-tagged PRC1. Movie duration is 46 hours. (time-lapse: 1 frame per 20 minutes).
(1.93 MB AVI)

Movie S2 Time-lapse movie of yellow fluorescence images of the clone with YFP CD-tagged PRC1. Movie duration is 46 hours. (time-lapse: 1 frame per 20 minutes).
(2.82 MB AVI)

Movie S3 Time-lapse movie of yellow fluorescence images overlaid on transmitted light images of the clone with YFP CD-tagged PRC1. Movie duration is 46 hours. (time-lapse: 1 frame per 20 minutes).
(2.03 MB AVI)

Movie S4 Time-lapse movie of transmitted light images of the clone with YFP CD-tagged YB1. Movie duration is 46 hours. (time-lapse: 1 frame per 20 minutes).
(2.25 MB AVI)

Movie S5 Time-lapse movie of yellow fluorescence images of the clone with YFP CD-tagged YB1. Movie duration is 46 hours. (time-lapse: 1 frame per 20 minutes).
(3.61 MB AVI)

Movie S6 Time-lapse movie of yellow fluorescence images overlaid on transmitted light images of the clone with YFP CD-tagged YB1. Movie duration is 46 hours. (time-lapse: 1 frame per 20 minutes).
(2.39 MB AVI)

Movie S7 Time-lapse movie of transmitted light images of the clone with YFP CD-tagged ANLN. Movie duration is 46 hours. (time-lapse: 1 frame per 20 minutes).
(1.60 MB AVI)

Movie S8 Time-lapse movie of yellow fluorescence images of the clone with YFP CD-tagged ANLN. Movie duration is 46 hours. (time-lapse: 1 frame per 20 minutes).
(1.12 MB AVI)

Movie S9 Time-lapse movie of yellow fluorescence images overlaid on transmitted light images of the clone with YFP CD-tagged ANLN. Movie duration is 46 hours. (time-lapse: 1 frame per 20 minutes).
(1.76 MB AVI)
