# Supplementary figures and images for: Protein Dynamics in Individual Human Cells: Experiment and Theory
Source: PLoS One. 2009 Apr 17;4(4):e4901. doi: 10.1371/journal.pone.0004901 (PMC2668709; doi:10.1371/journal.pone.0004901)

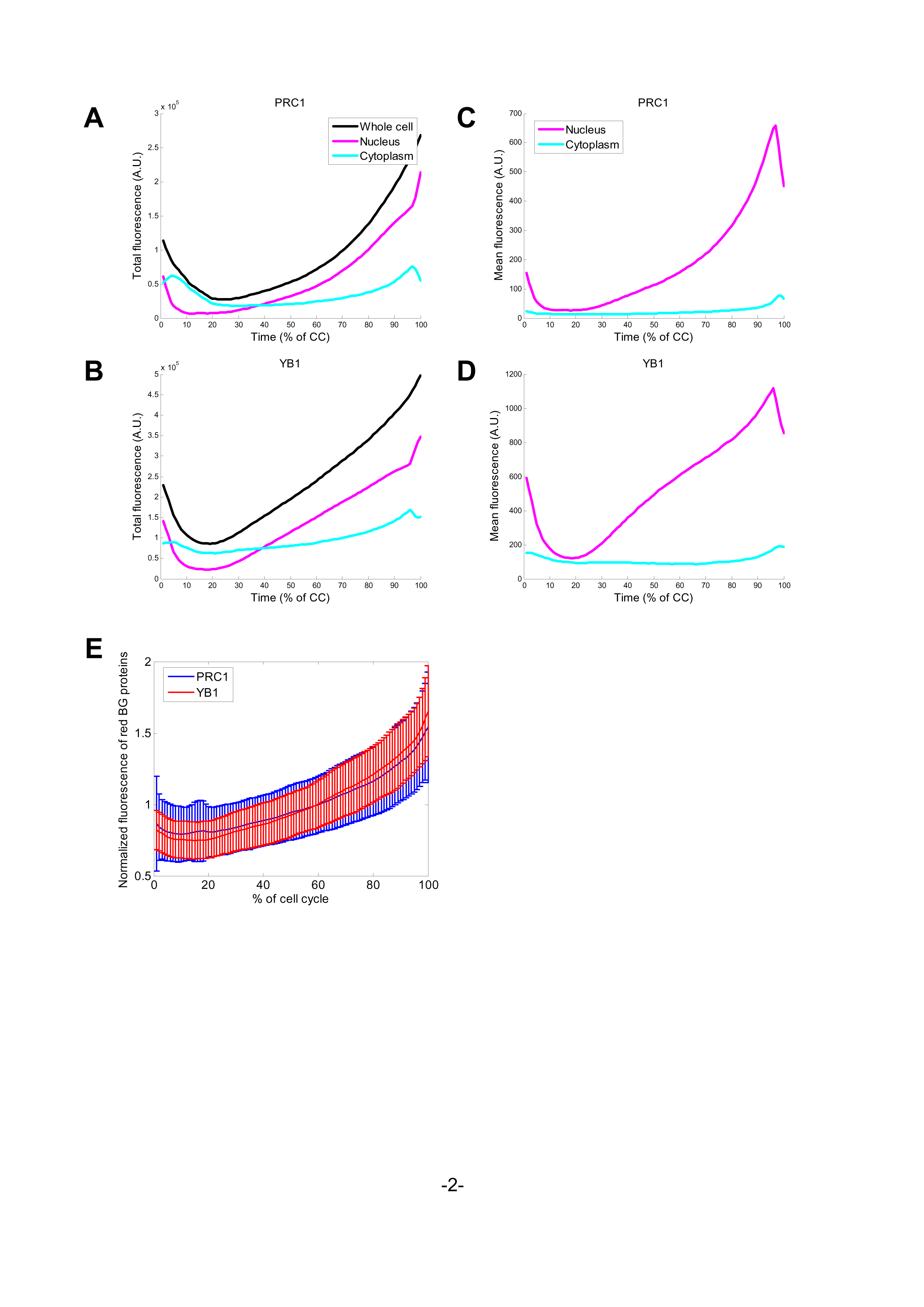

Supplement: Figure S1 — Immunoblots of YB1, PRC1, and ANLN with anti-GFP. Estimated molecular weight of each protein without yellow fluorescent tag is: YB1 ∼36 kDa, PRC1 ∼71 kDa and ANLN ∼124 kDa. YFP ∼27 kDa. (1.85 MB TIF) [file pone.0004901.s001.tif]

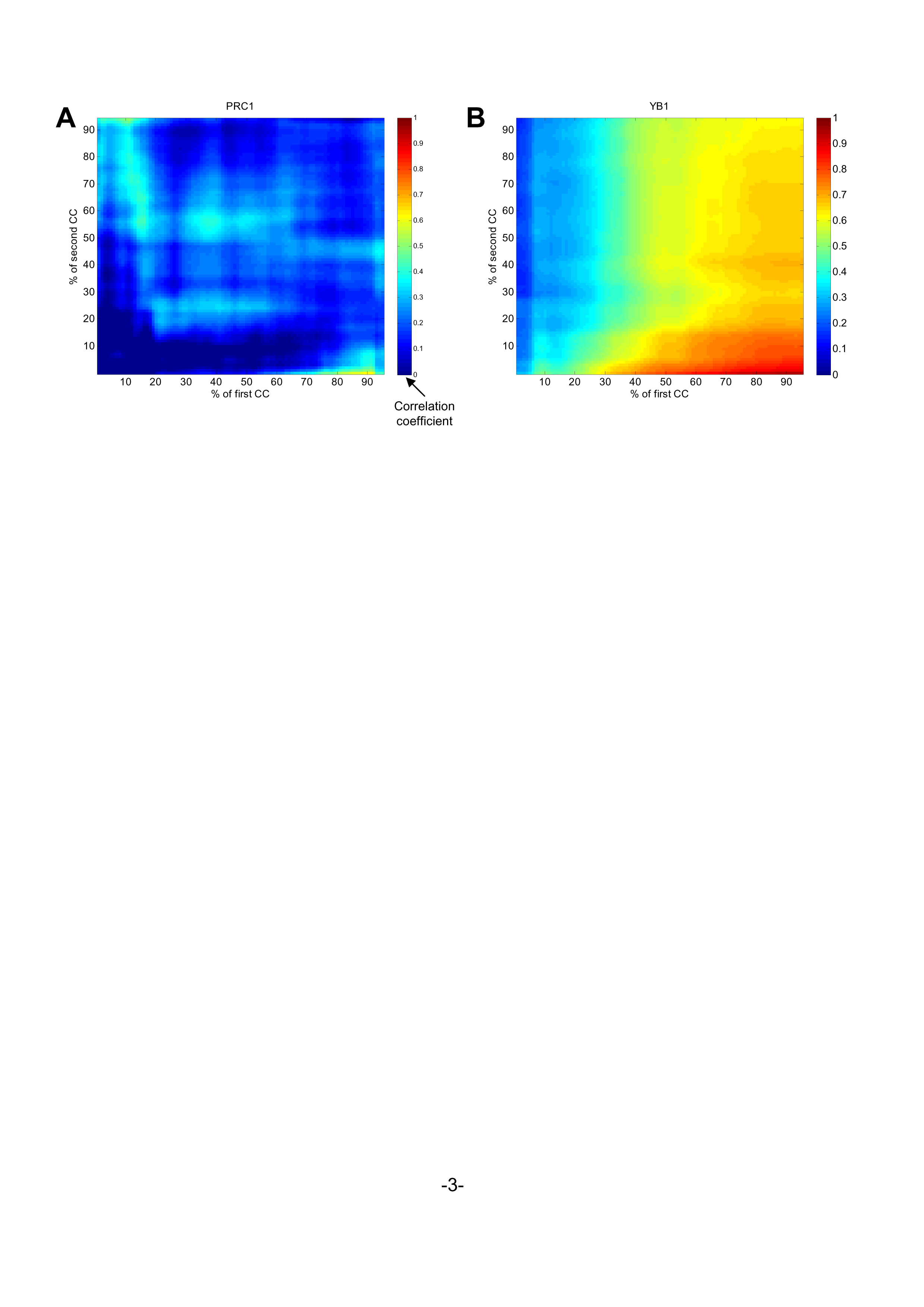

Supplement: Figure S3 — Correlation across consecutive cell cycles. (A,B) cells were tracked for two consecutive cell cycles. Shown is the correlation matrix of each time point in first cell cycle and all time points in the second cell cycle for PRC1 and YB1 respectively. Note that PRC1 doesn't retain any memory across cell cycles while YB1 shows relatively high correlation between fluorescent levels of a mother cell and fluorescent levels in daughter cell. (1.73 MB TIF) [file pone.0004901.s003.tif]

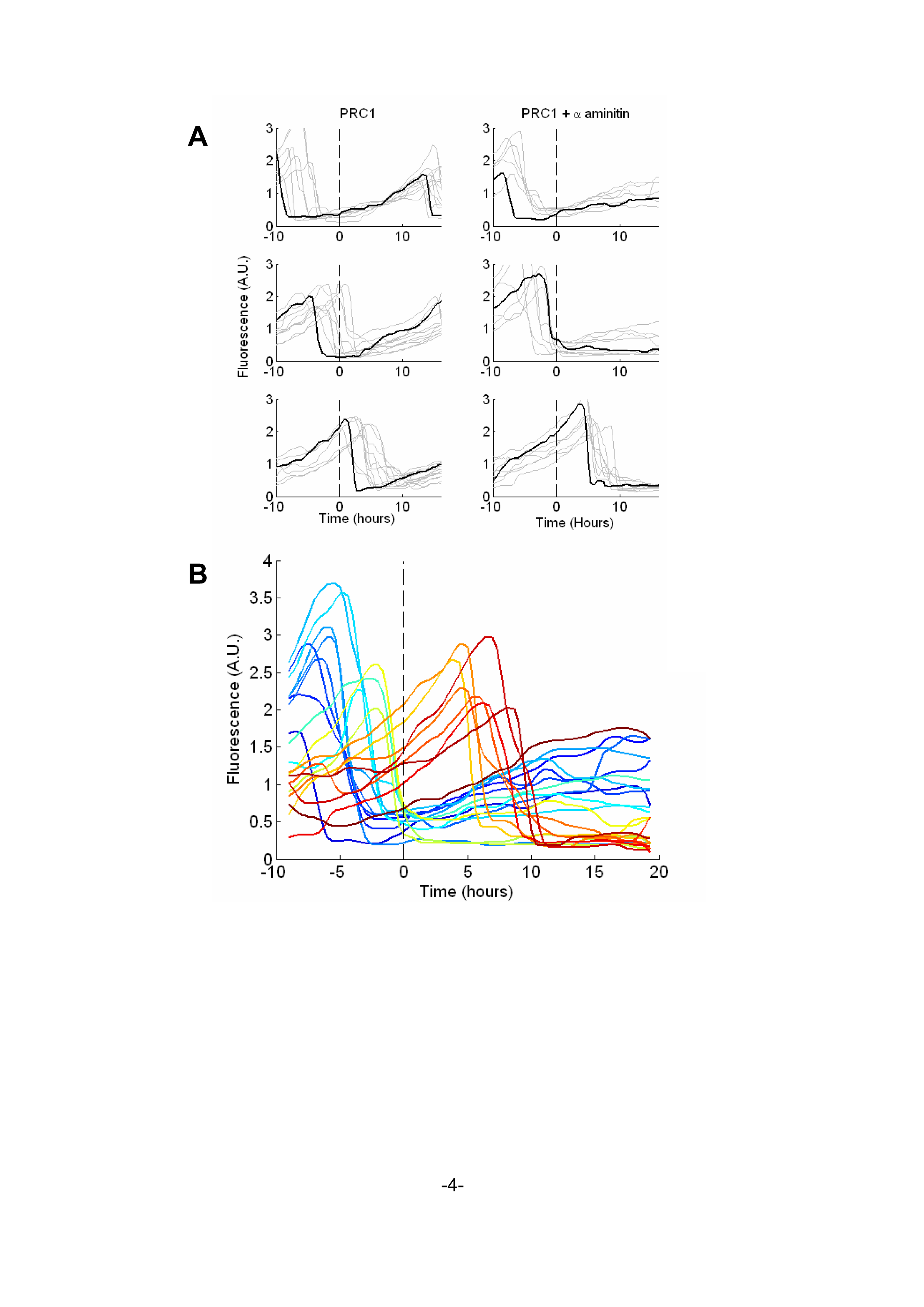

Supplement: Figure S4 — Response of single cells expressing tagged PRC1 at different stages of their cell cycle to transcription inhibitor reveal mRNA degradation at beginning of cell cycle. (A) Cells were divided into three groups based on the time of their division relative to the time of administration of α-aminitin, a transcription inhibitor (time = 0 hrs). Top group - division before drug addition, middle group - division during drug addition and bottom group - division after drug addition. Left panel - 2 ml medium was exchanged with 2 ml fresh medium (at t = 0 hrs), right panel - 2 ml medium was exchanged with 2 ml fresh medium containing 100 µg/ml α-aminitin (at t = 0 hrs). Each grey line denotes measurements of a single cell, black line is a chosen cell for eye guidance. Note that as cell divide later in the experiment the slope of protein levels decreases. (B) all trajectories of right panel overlaid and color ordered based on time of cell division, early divisions in blue and late divisions in red. (2.21 MB TIF) [file pone.0004901.s004.tif]

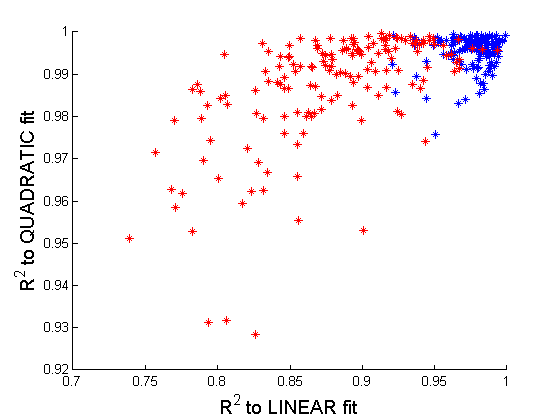

Supplement: Figure S5 — Single cell trajectories display linear profiles in the case of YB1 and quadratic profiles in the case of PRC1. Each single cell trajectory denoting fluorescent levels measured over the cell cycle was fit to a polynomial function of first and second degree. Plotted are the pearson correlation coefficient values (R2) of the experimental data and the fit for PRC1 (red) and YB1 (blue). All trajectories of YB1 showed R2>0.9 already in the linear fit, while PRC1 showed R2>0.9 for all cells only when using a polynomial of 2nd degree. (0.71 MB TIF) [file pone.0004901.s005.tif]

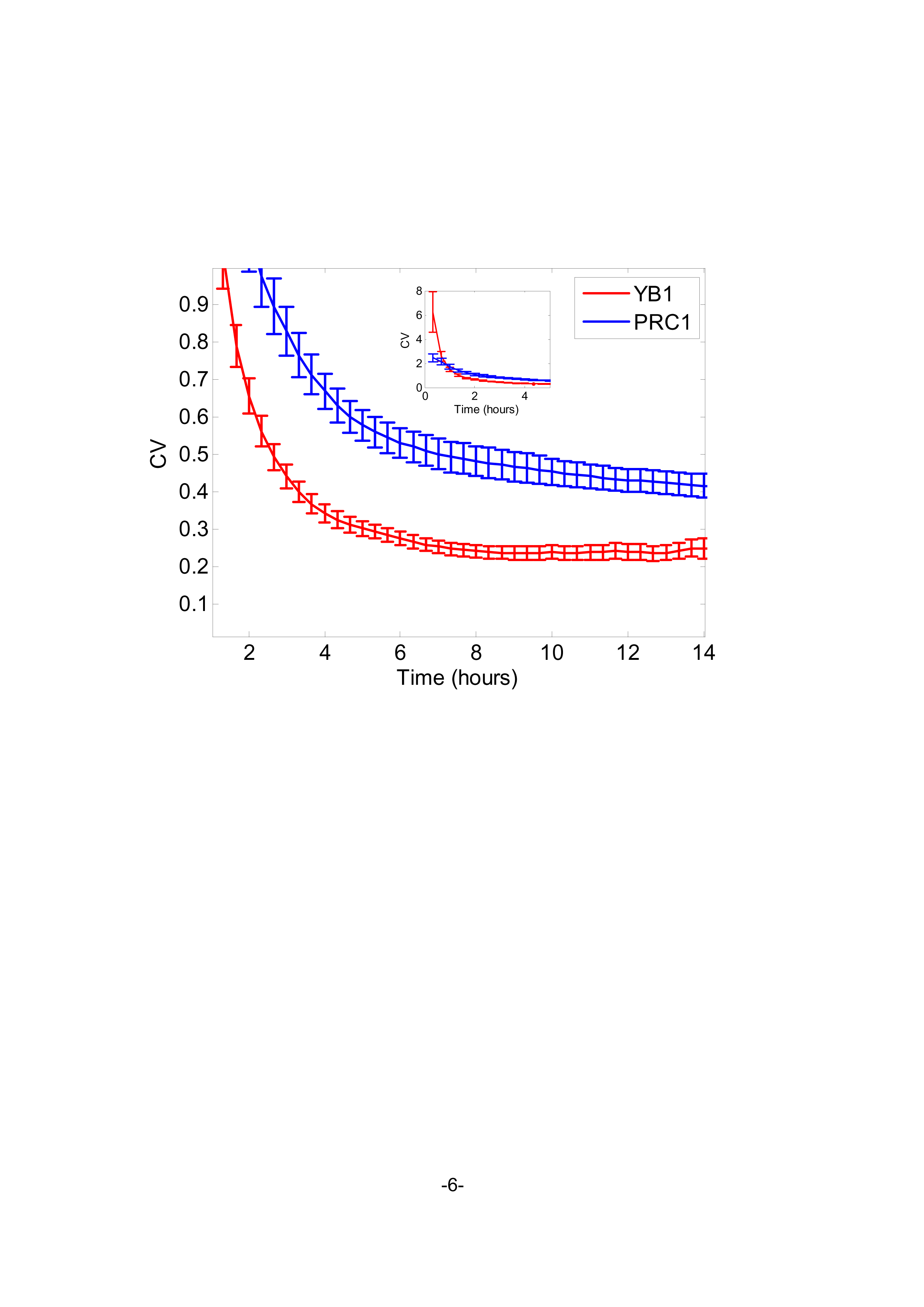

Supplement: Figure S6 — Coefficient of Variance (CV) of PRC1 and YB1. CV of PRC1 (blue) and YB1 (red) of protein levls across cells synchronized to the beginning of protein accumulation. Inset denotes the CV of the first 4 hours. (1.35 MB TIF) [file pone.0004901.s006.tif]

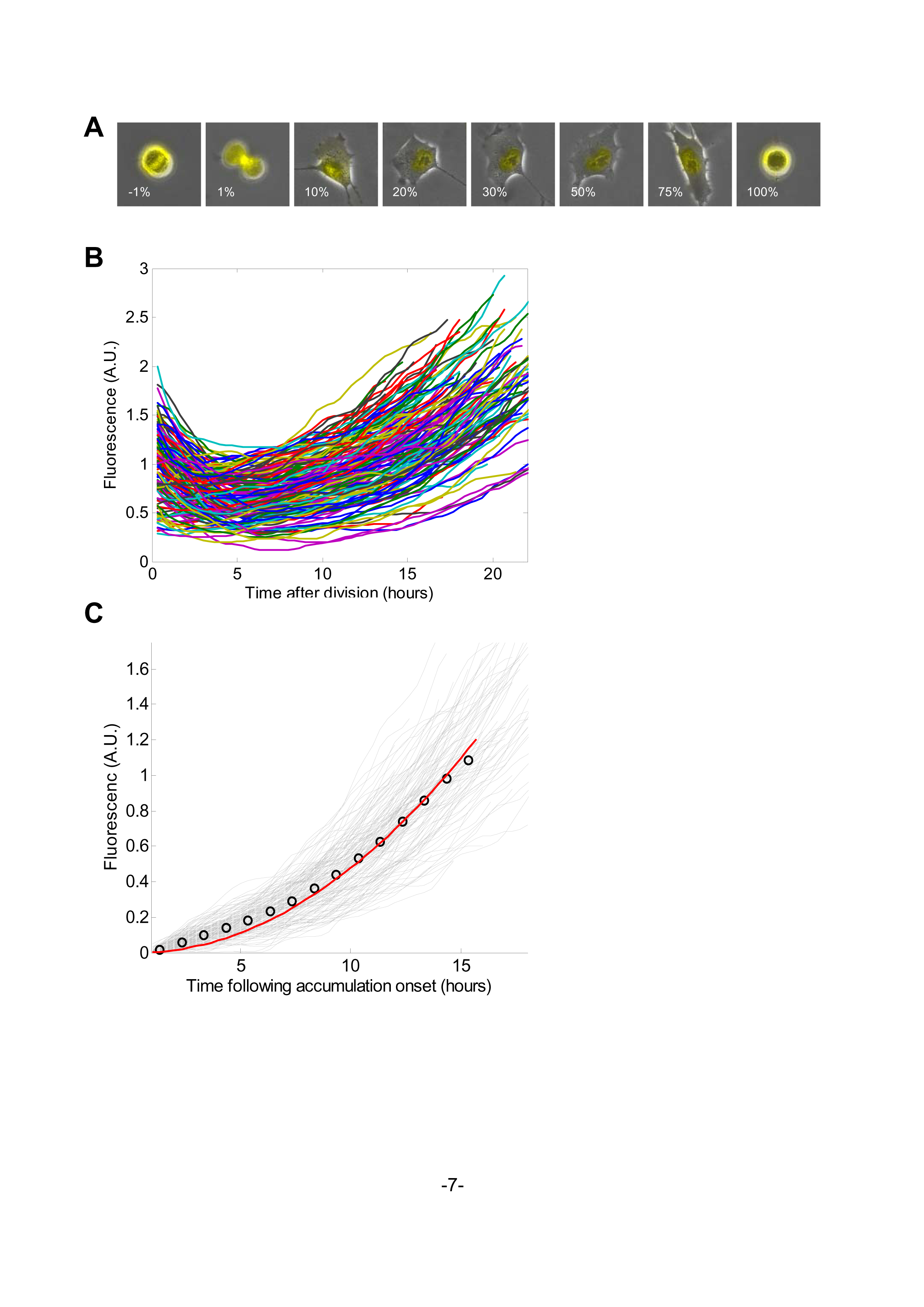

Supplement: Figure S7 — Cell cycle dependent behavior of ANLN. (A) Example of one cell automatically tracked through the cell cycle. Each image is an overlay of fluorescent tagged protein on phase contrast image. Images are ordered according to the fraction of the time elapsed between two division events. The cells are automatically centered. The percentage of elapsed cell cycle is indicated at the bottom, Bar, 10 µm. Note asymmetric division of fluorescently tagged PRC1 between the two daughter cells following cell division. (B,C) For each protein, PRC1 and YB1 tracks of 150 cells are plotted. Each line denotes the total fluorescence level as measured during the cell cycle of a single cell. Cells are synchronized to beginning of cell cycle. Each cell has different cell cycle length (PRC1: μ = 21.6±2 hrs, YB1: 17.2±2 hrs). Both proteins are degraded following cell division. (1.52 MB TIF) [file pone.0004901.s007.tif]

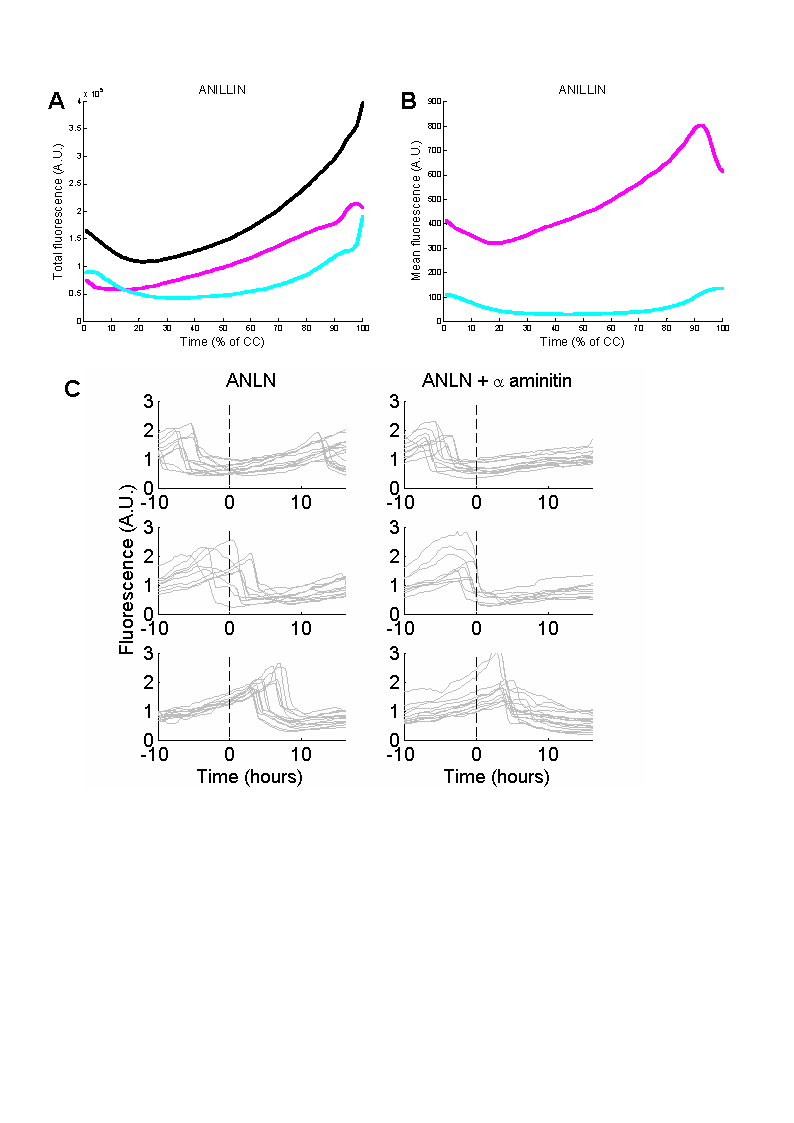

Supplement: Figure S8 — Cell cycle dependent behavior of ANLN. (A) Mean of total fluorescent levels in different compartments of the cell (cyan - cytoplasm, magenta - nucleus and black- whole cell). (B) Mean of mean fluorescent levels in different compartments of the cell (cyan - cytoplasm, magenta - nucleus). (C) The same as Legend of figure S5A just for ANLN. (0.10 MB TIF) [file pone.0004901.s008.tif]
