# Supplementary material for: Protein Dynamics in Individual Human Cells: Experiment and Theory
Source: PLoS One. 2009 Apr 17;4(4):e4901. doi: 10.1371/journal.pone.0004901 (PMC2668709; doi:10.1371/journal.pone.0004901)
Supplement: Text S1 — (0.61 MB PDF) [file pone.0004901.s009.pdf]

Supporting Material

Supporting text

- **Part 1 - General properties of cell cycle behavior of PRC1 and YB1 (pg. 1-3)**
- **Part 2 - Fitting parameters to the transcription inhibition experiment (pg. 4-5)**
- **Part 3 - A simple stochastic model describing transcription and translation (pg. 6-11)**
- **Part 4 - A model including stochastic gene activation and inactivation (pg. 12-17)**
- **Part 5 - Analytical solution for time-dependent moments (pg. 18-20)**
- **Part 6 - Protein-level distribution (pg. 21-22)**
- **Part 7 - Distribution of onset-times (pg. 23-25)**
- **Part 8 - Estimation of translation rates (pg. 26)**
- **Part 9 - Extraction of biochemical parameters - fitting (pg. 27-31)**
- **Part 10 - Comparison of extrinsic and intrinsic simulations (pg. 32-35)**

Supporting movies (pg. 36)

Supporting references (pg. 37)

General properties of cell cycle behavior of PRC1 and YB1

The cell-cycle time is 21.6 ± 2 hrs for PRC1 and 17.2 ± 2 hrs for YB1 clones. We measured the correlation between fluorescence levels of the tagged proteins PRC1 and YB1 at the end of the cell cycle and several cellular factors such as cell cycle length, initial protein levels and cell size. For PRC1 we found low correlation with cell size ($R = 0.21$ $p<0.05$) and no correlation with cell cycle length ($R = 0.02$ $p = 0.8$) or with initial fluorescence levels ($R = 0.09$ $p = 0.2$). However, YB1 showed opposite tendencies: no correlation with cell size ($R = 0.08$ $p = 0.3$) and moderate correlation with cell cycle length ($R = 0.44$ $p<0.05$) and initial fluorescence levels ($R = 0.5$ $p<0.05$) (Figure S8A-F). When no correlation is found it suggests that different aspects of the biological phenomena are independent of each other. On the contrary, when correlation is found, it may suggest causative connections between processes at different stages of the cell cycle and the protein level at the end of the cell cycle.

We found strong correlation ($R=0.99$ for PRC1 and $R=0.9$ for YB1, $p<0.05$) between initial protein levels inherited by each daughter cell and the absolute amount of protein degraded at the beginning of the cell cycle (Figure S9A,B). In the case of PRC1 this correlation is due to the fact that almost all initial protein is degraded.

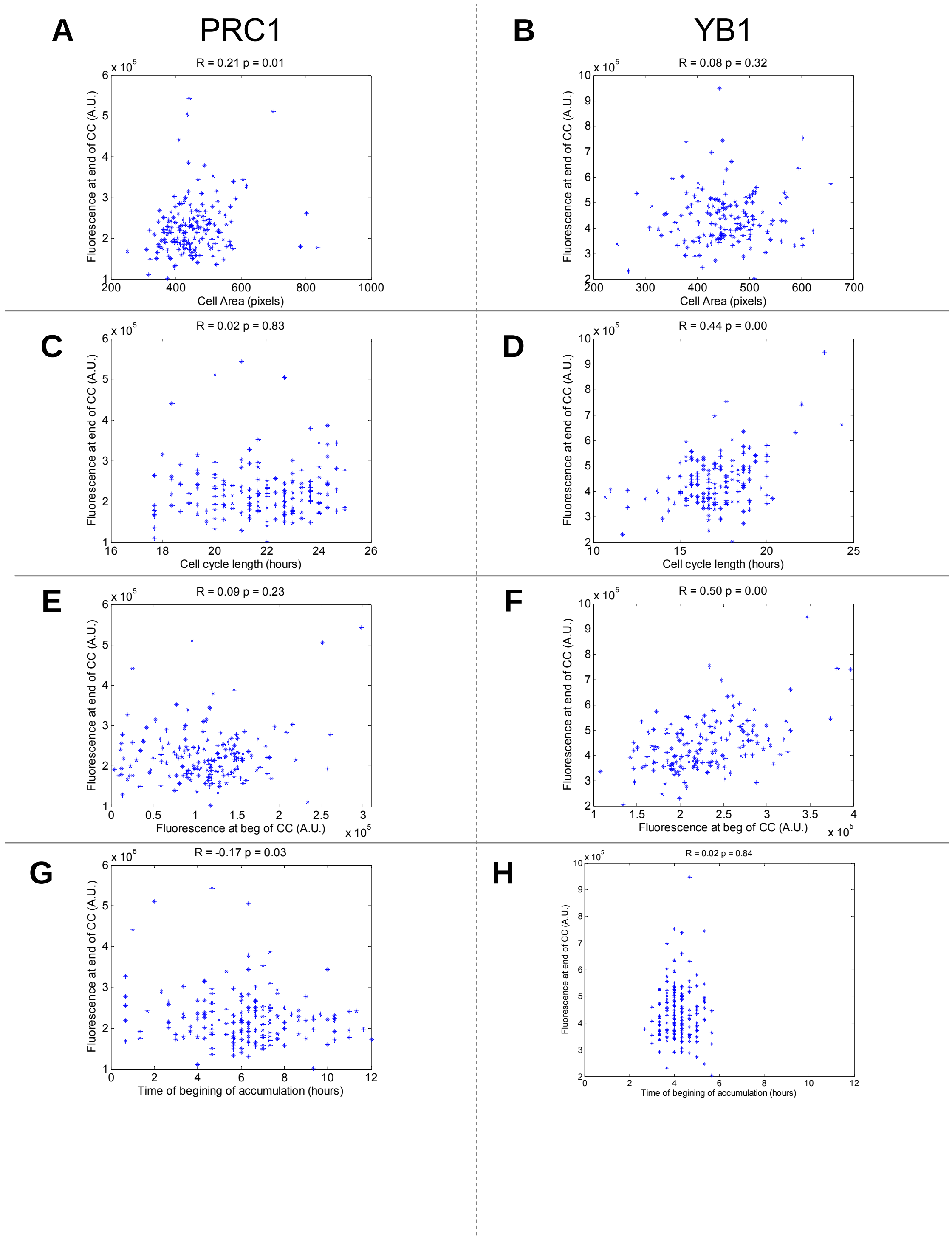

Figure S8: Correlation of fluorescence levels at end of cell cycle with 1. cell area at end of cell cycle (A-B), 2. cell cycle length (C-D), 3. fluorescence at beginning of cell cycle (E-F) and 4. Time of beginning of accumulation (G-H) for PRC1 and YB1 respectively. For PRC1 no significant correlation was found with any of the above four parameters. YB!, showed significant correlation between protein levels at end of cell cycle and cell cycle age (D) and fluorescence at beginning of cell cycle (F).

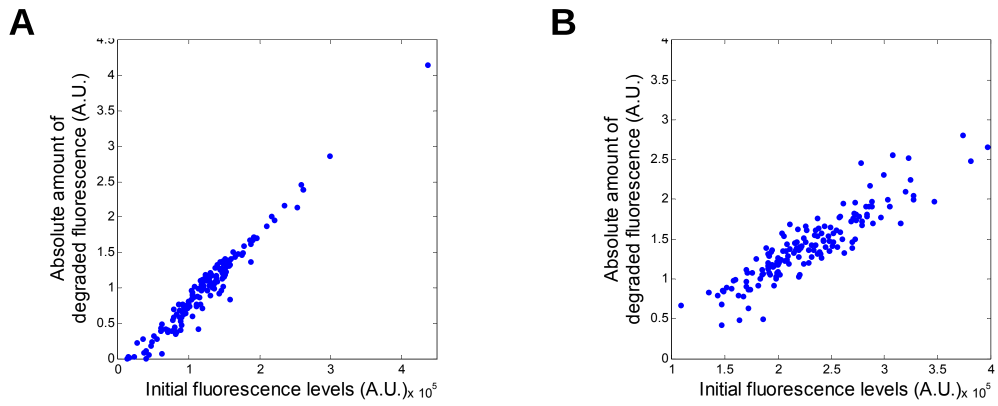

Figure S9: Correlation across consecutive cell cycles. A,B) Correlation between fluorescent levels of tagged protein at the beginning of cell cycle and the absolute amount of degradation (measured as difference between initial levels and minimum levels) for PRC1 and YB1 respectively

Fitting parameters to the transcription inhibition experiment:

A transcription inhibitor is added to the cell clones expressing YB1 at $t = 0\,\text{hr}$. If the inhibitor starts taking affect at time t_0 then the differential equations for mRNA levels $r(t)$ and protein levels $p(t)$ are:

$$\frac{dr}{dt} = k_R P_1 - \alpha_R r \tag{S 1}$$

$$\frac{dp}{dt} = k_P r \tag{S 2}$$

where $k_R = 0$ for $t > t_0$. The solution to these equations is detailed in Table S1:

Table S1: Analytical solution for the dynamics of the mean mRNA and protein levels for the inhibition experiment done on YB1 reporter clones.

$1/\alpha_R \ll t \le t_0$	$t > t_0$
$r(t) = \frac{k_R P_1}{\alpha_R}$<br>$p(t) = \frac{k_R k_P P_1}{\alpha_R} \cdot \left(t - \frac{1}{\alpha_R} \right) + p(0)$	$r(t) = \frac{k_R P_1}{\alpha_R} \cdot e^{-\alpha_R (t - t_0)}$<br>$p(t) = \frac{k_R k_P P_1}{\alpha_R} \cdot \left(\frac{1 - e^{-\alpha_R (t - t_0)}}{\alpha_R} \right) + p(t_0)$

A good fit is achieved for $t_0 = 2.33\,\text{hr}$, $\alpha_R = 0.77\,\text{hr}^{-1}$ as shown in Figure S8. This conforms to previous observations which indicate that the inhibitor starts taking affect approximately 2 hours after introduction.

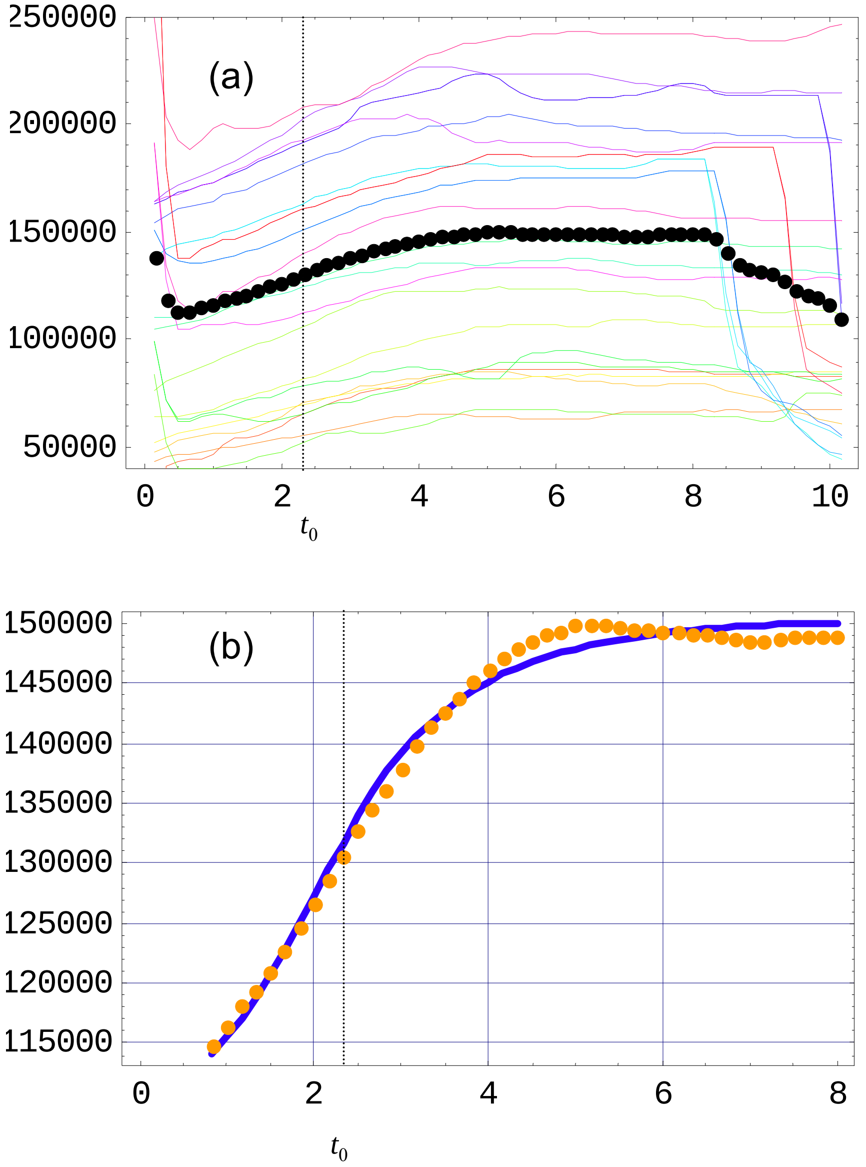

Figure S8: Fitting the mean protein levels for the transcription inhibition experiment. Shown are the YB1 protein levels in individual cells (a), their mean (orange circles) (b). Fitting to the analytical solution (blue line) indicates that the inhibitor starts taking affect roughly $t_0 = 2.33$ hours after introduction into the medium in line with Nyugen *et al.* (Nguyen, Giannoni et al. 1996).

A simple stochastic model describing transcription and translation:

General description:

Both PRC1 and YB1 can be described by a simple stochastic model for gene expression. The model assumes that mRNA is produced at rate k_R, and degraded with degradation rate α_R (for PRC1 we assume that the mRNA is stable and take $\alpha_R \approx 0$). Protein is produced at rate k_P from each molecule of mRNA, and is taken to be stable throughout the cell cycle. The cell cycle is approximately 20 hours.

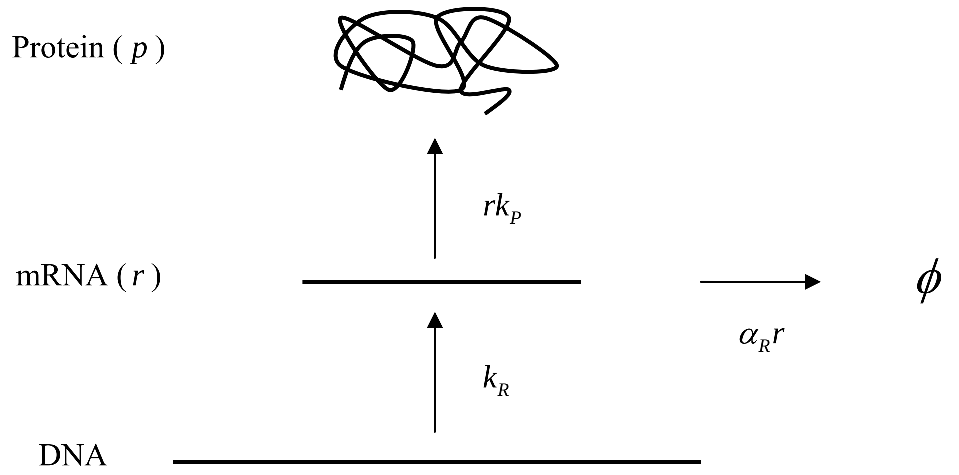

Figure S9: A sketch of the stochastic model for transcription and translation.

The master equation:

Let $f_{r,p}$ represent the relative number of cells with p copies of protein and r mRNA molecules [i.e. the probability to be in state (r,p)]. The transitions between the different states may be described by Figure S10. The dynamics of $f_{r,p}$ is described by the following master equation:

$$\frac{df_{r,p}}{dt} = -k_R f_{r,p} - k_P r f_{r,p} + k_R f_{r-1,p} + k_P r f_{r,p-1} + \alpha_R (r+1) f_{r+1,p} - \alpha_R r f_{r,p} \qquad \textbf{(S 3)}$$

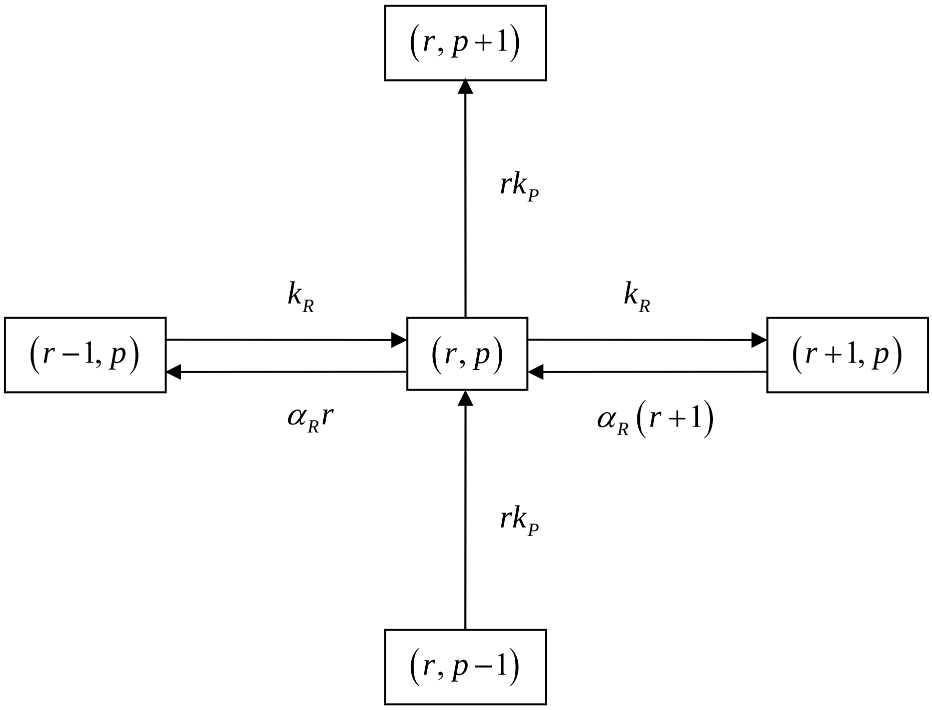

Figure S10: State diagram of the stochastic model for transcription and translation. 6 arrows correspond to 6 terms: 3 incoming (+) and 3 outgoing (-). Note that r cannot be negative due to the fact that the degradation from state $r=0$ is proportional to $\alpha_R r=0$.

The master equation can be solved analytically for the mean and variance (and all higher moments) of the protein and mRNA levels. Using the generating function $F(x,y)$ and its derivatives with respect to x and y (as detailed in Table S2) the master equation can be written as:

$$\dot{F}=-k_R F-k_P x\frac{\partial F}{\partial x}+k_R xF+k_P xy\frac{\partial F}{\partial x}+\alpha_R\frac{\partial F}{\partial x}-\alpha_R x\frac{\partial F}{\partial x} \qquad \textbf{(S 4)}$$

Substituting $F(x,y)=\sum_{r,p=1}^{\infty} f_{r,p}x^r y^p$ and comparing coefficients of $x^r y^p$ from both sides of the equation, it is possible to obtain differential equations for the time-dependent moments $\langle r\rangle$, $\langle p\rangle$, $\langle rp\rangle$, $\langle r^2\rangle$, and $\langle p^2\rangle$. This is actually done by differentiating the master equation with increasing orders of x and y, and substituting $x=y=1$, see Table S3.

Table S2: The generating function of $f_{r,p}$ and all its derivatives.

Generating function	Coefficient of $x^r y^p$	Substitution of $x = y = 1$
$F(x,y) = \sum_{r,p=0}^{\infty} f_{r,p} x^r y^p$	$f_{r,p}$	$F(1,1) = \sum_{r,p=0}^{\infty} f_{r,p} = 1$
$\dot{F} = \sum_{r,p=0}^{\infty} \dot{f}_{r,p} x^r y^p$	$\frac{df_{r,p}}{dt}$	$\dot{F}\Big\vert_{1,1} = \sum_{r,p=0}^{\infty} \dot{f}_{r,p} = \frac{d}{dt}\sum_{r,p=0}^{\infty} f_{r,p} = \frac{d}{dt}(1) = 0$
$\frac{\partial F}{\partial x} = \sum_{r,p=0}^{\infty} f_{r,p} r x^{r-1} y^p$	$(r+1) f_{r+1,p}$	$\frac{\partial F}{\partial x}\Big\vert_{1,1} = \sum_{r,p=0}^{\infty} f_{r,p} r = \langle r \rangle$
$\dot{\left(\frac{\partial F}{\partial x}\right)} = \sum_{r,p=0}^{\infty} \dot{f}_{r,p} r x^{r-1} y^p$		$\dot{\left(\frac{\partial F}{\partial x}\Big\vert_{1,1}\right)} = \dot{\left(\sum_{r,p=0}^{\infty} f_{r,p} r\right)} = \langle \dot{r} \rangle$
$x\frac{\partial F}{\partial x} = \sum_{r,p=0}^{\infty} f_{r,p} r x^r y^p$	$r f_{r,p}$	$x\frac{\partial F}{\partial x}\Big\vert_{1,1} = \sum_{r,p=0}^{\infty} f_{r,p} r = \langle r \rangle$
$xF = \sum_{r,p=0}^{\infty} f_{r,p} x^{r+1} y^p$	$f_{r-1,p}$	$xF\Big\vert_{1,1} = \sum_{r,p=0}^{\infty} f_{r,p} = 1$
$xy\frac{\partial F}{\partial x} = \sum_{r,p=0}^{\infty} f_{r,p} r x^r y^{p+1}$	$r f_{r,p-1}$	$xy\frac{\partial F}{\partial x}\Big\vert_{1,1} = \sum_{r,p=0}^{\infty} f_{r,p} r = \langle r \rangle$
$\frac{\partial^2 F}{\partial x^2} = \sum_{r,p=0}^{\infty} f_{r,p} r(r-1) x^{r-2} y^p$		$\frac{\partial^2 F}{\partial x^2} = \sum_{r,p=0}^{\infty} f_{r,p} r(r-1) = \langle r(r-1) \rangle = \langle r^2 \rangle - \langle r \rangle$
$\dot{\left(\frac{\partial^2 F}{\partial x^2}\right)} = \sum_{r,p=0}^{\infty} \dot{f}_{r,p} r(r-1) x^{r-2} y^p$		$\dot{\left(\frac{\partial^2 F}{\partial x^2}\Big\vert_{1,1}\right)} = \dot{\left(\langle r^2 \rangle - \langle r \rangle\right)}$
$\frac{\partial^2 F}{\partial x \partial y} = \sum_{r,p=0}^{\infty} r p f_{r,p} x^{r-1} y^{p-1}$		$\frac{\partial^2 F}{\partial x \partial y}\Big\vert_{1,1} = \sum_{r,p=0}^{\infty} r p f_{r,p} = \langle rp \rangle$

Table S3: Equations for time-dependent moments $\langle r\rangle$, $\langle p\rangle$, $\langle r^2\rangle$, and $\langle p^2\rangle$.

	Equation	How to derive this equation from the master equation.
1	$\dot{\langle r\rangle} = k_R - \alpha_R \langle r\rangle$	Differentiate the master equation with respect to x, and substitute $x = y = 1$.
2	$\dot{\left(\langle r^2\rangle - \langle r\rangle\right)} = 2k_R \langle r\rangle - 2\alpha_R \left(\langle r^2\rangle - \langle r\rangle\right)$	Differentiate twice with respect to x, etc.
3	$\dot{\langle p\rangle} = k_P \langle r\rangle$	Differentiate with respect to y.
4	$\dot{\left(\langle p^2\rangle - \langle p\rangle\right)} = 2k_P \langle rp\rangle$	Differentiate twice with respect to y.
5	$\dot{\langle rp\rangle} = k_R \langle p\rangle + k_P \langle r^2\rangle - \alpha_R \langle rp\rangle$	Differentiate with respect to x and then y.

Analytical solution for time-dependent moments:

Solving the differential equations in Table S3 for YB1 ($\alpha_R > 0$) and PRC1 ($\alpha_R = 0$), we obtain the time-dependent mean and variance of protein and mRNA in the cell population. From these quantities it is possible to calculate the Noise strength $\mathrm{NS}(p) = \langle \Delta p^2\rangle / \langle p\rangle$ and Coefficient of variance $\mathrm{CV}(p) = \langle \Delta p\rangle / \langle p\rangle$ of protein levels, as shown in Table S4. It can be seen that asymptotically (i.e., for $t \gg 1/\alpha_R$), the mean protein level of YB1 is increasing linearly with time ($\langle p\rangle \sim t$), whereas for PRC1 it grows quadratically ($\langle p\rangle \sim t^2$).

For Poissonian processes, the variance is equal to the mean, and Noise strength is equal to 1. In our case we define a parameter b to quantify the deviation from Poisson such that $\langle \Delta p^2\rangle / \langle p\rangle \equiv 1 + b$. From Table S4 it is seen that for YB1 the non-Poissonnian parameter is asymptotically constant, $b = 2k_P / \alpha_R$, whereas for PRC1 it grows linearly with time: $b = (2k_P / 3)\cdot t$. The CV however, is asymptotically decreasing with time for both cases: $\langle \Delta p\rangle / \langle p\rangle \sim 1/\sqrt{t}$.

Using the expressions for the mean protein levels $\langle p\rangle$, the Noise strength, and the CV, it is possible in principle to estimate the biochemical parameters k_R, α_R, and k_P from the experimental data of protein levels in single cells. It is important to note that when measuring the protein levels using fluorescent reporters, the mean number of proteins and the noise strength are multiplied by a proportionality factor which represents the number of fluorescent units per protein molecule. This factor has to be measured independently. However, the CV is invariant to measurement units.

Table S4: Mean time-dependent levels of protein $\langle p \rangle$ and mRNA $\langle r \rangle$, and their respective noise strengths. For YB1 the asymptotic behavior of the mean protein level is increasing linearly with time ($\langle p \rangle \sim t$), whereas for PRC1 it grows quadratically ($\langle p \rangle \sim t^2$). The non-Poissonian parameter b is asymptotically constant for YB1, whereas it grows linearly for PRC1. For both cases the CV of the protein level is asymptotically decreasing with time as: $\langle \Delta p \rangle / \langle p \rangle \sim 1/\sqrt{t}$.

	**YB1**<br>Short mRNA lifetime	**PRC1**<br>Stable mRNA ($\alpha_R \to 0$)
Mean $\langle r \rangle$	$\frac{k_R}{\alpha_R}\left(1-e^{-\alpha_R t}\right)$<br>For long times: $\frac{k_R}{\alpha_R}$	$k_R t$
Mean $\langle p \rangle$	$k_P \frac{k_R}{\alpha_R}\left(t+\frac{e^{-\alpha_R t}-1}{\alpha_R}\right)$<br>For long times: $k_P \frac{k_R}{\alpha_R}\cdot t$	$\frac{k_P k_R}{2}\cdot t^2$
Noise strength $\frac{\langle \Delta r^2 \rangle}{\langle r \rangle}$	1	1
Noise strength (asymptotically): $\frac{\langle \Delta p^2 \rangle}{\langle p \rangle} \equiv 1+b$	$1+\frac{2k_P}{\alpha_R}$	$1+\frac{2k_P}{3}\cdot t$

Simulation:

In order to simulate the stochastic process we use the Gillespie algorithm:

1. Define the *species*: r, p.
2. Define the *reactions*:
   (i) Production of mRNA
   (ii) Degradation of mRNA
   (iii) Production of protein.
3. Initialize: $t = 0$, $r = r_0$, $p = p_0$.
4. <u>For each iteration</u>:
   a. Calculate *probabilities* for all *reactions:* $a_1 = k_R$, $a_2 = r\alpha_R$, $a_3 = rk_P$, and the total probability: $a_0 = a_1 + a_2 + a_3$.

b. Calculate the time T for next reaction, and the next reaction m. The basic assumption is that the probability that reaction m will occur after time interval T is: $P(T,m) = a_m e^{-a_0 T}$.
   (i) Choose two random numbers: $r_1, r_2 \in [0,1]$,
   (ii) Time for next reaction: Solve $r_1 = e^{-a_0 T}$ for T.
   (iii) Which is the next reaction?
   Find m such that: $a_1 + ... + a_{m-1} < r_2 a_0 < a_1 + ... + a_{m-1} + a_m$.

c. Update:
   (i) Update time: $t \rightarrow t + T$.
   (ii) Execute reaction m. See Table S4

Table S5: The reactions and their probabilities as implemented by the Gillespie algorithm for simulating PRC1 and YB1 gene expression.

m	Probability	Reaction implementation
1	$a_1 = k_R$	$r \rightarrow r+1$
2	$a_2 = r\alpha_R$	$r \rightarrow r-1$
3	$a_3 = rk_P$	$p \rightarrow p+1$

A model including stochastic gene activation and inactivation

General description:

A more complete description of gene expression in eukaryotic cells should take into account the stochastic process of gene activation and deactivation. These fluctuations occur due to random events of binding and unbinding of the DNA to the chromatin ("chromatin remodeling"). The gene is active and mRNA can be produced only when the DNA is "open", i.e. not bound tightly to the chromatin – see figure S11. We assume that the transitions between active and inactive states occur at rates k_{on} and k_{off}, and are rather slow (a few events per hour). Mathematically this process is known as a "Telegraph process".

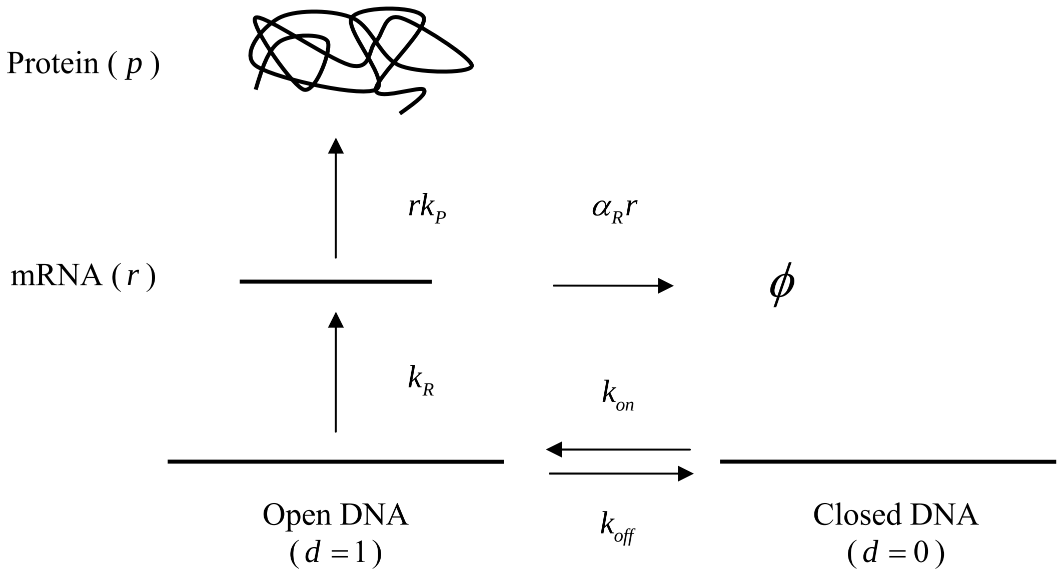

Figure S11: A sketch of the stochastic model for gene expression, including the relatively slow process of gene activation and inactivation. The transition between open and closed conformations occurs at rates k_{off} and k_{on}.

The master equation:

Let $f_{r,p,1}$ represent the relative number of cells with open DNA, p copies of protein, and r mRNA molecules, and let $f_{r,p,0}$ represent the relative number of cells with same parameters but closed DNA [i.e. $f_{r,p,d}$ is the probability to be in state (r, p, d) where $d \in \{0,1\}$].

The transitions between the different states are shown in Figure S12. The dynamics of $f_{r,p,d}$ is described by *two* master equations: one for $d=0$ (inactive state) and one for $d=1$ (active state):

$$\frac{df_{r,p,1}}{dt} = -k_R f_{r,p,1} - k_P r f_{r,p,1} + k_R f_{r-1,p,1} + k_P r f_{r,p-1,1} + \alpha_R (r+1) f_{r+1,p,1} - \alpha_R r f_{r,p,1} + \ldots \\ \ldots + k_{on} f_{r,p,0} - k_{off} f_{r,p,1} \quad \textbf{(S 5)}$$

$$\frac{df_{r,p,0}}{dt} = \qquad -k_P r f_{r,p,0} \qquad + k_P r f_{r,p-1,0} + \alpha_R (r+1) f_{r+1,p,0} - \alpha_R r f_{r,p,0} + \ldots \\ \ldots + k_{off} f_{r,p,1} - k_{on} f_{r,p,0} \quad \textbf{(S 6)}$$

These coupled equations can be solved analytically for the mean and variance (and all higher moments) of the protein levels p and mRNA levels r, as well as for the gene activity d.

For this model we use *two* generating functions (Peccoud and Ycart 1995) that correspond to the two states $f_{r,p,1}$ and $f_{r,p,0}$ (see Table S6):

$$F_1(x,y) = \sum_{r,p=0}^{\infty} f_{r,p,1} x^r y^p \quad \textbf{(S 7)}$$

$$F_0(x,y) = \sum_{r,p=0}^{\infty} f_{r,p,0} x^r y^p \quad \textbf{(S 8)}$$

Using the above formalism, the two master equations can be written as:

$$\dot{F}_1 = -k_R F_1 - k_P x \frac{\partial F_1}{\partial x} + k_R x F_1 + k_P xy \frac{\partial F_1}{\partial x} + \alpha_R \frac{\partial F_1}{\partial x} - \alpha_R x \frac{\partial F_1}{\partial x} + k_{on} F_0 - k_{off} F_1 \quad \textbf{(S 9)}$$

$$\dot{F}_0 = \qquad - k_P x \frac{\partial F_0}{\partial x} \qquad + k_P xy \frac{\partial F_0}{\partial x} + \alpha_R \frac{\partial F_0}{\partial x} - \alpha_R x \frac{\partial F_0}{\partial x} + k_{off} F_1 - k_{on} F_0 \quad \textbf{(S 10)}$$

Again, substituting $F_d(x,y) = \sum_{r,p=0}^{\infty} f_{r,p,d} x^r y^p$ and comparing coefficients of $x^r y^p$ from both sides of the equation, it is possible to obtain differential equations for the time-dependent moments $\langle d \rangle$, $\langle d^2 \rangle$, $\langle r \rangle$, $\langle p \rangle$, $\langle rp \rangle$, $\langle r^2 \rangle$, and $\langle p^2 \rangle$ - see Table S7.

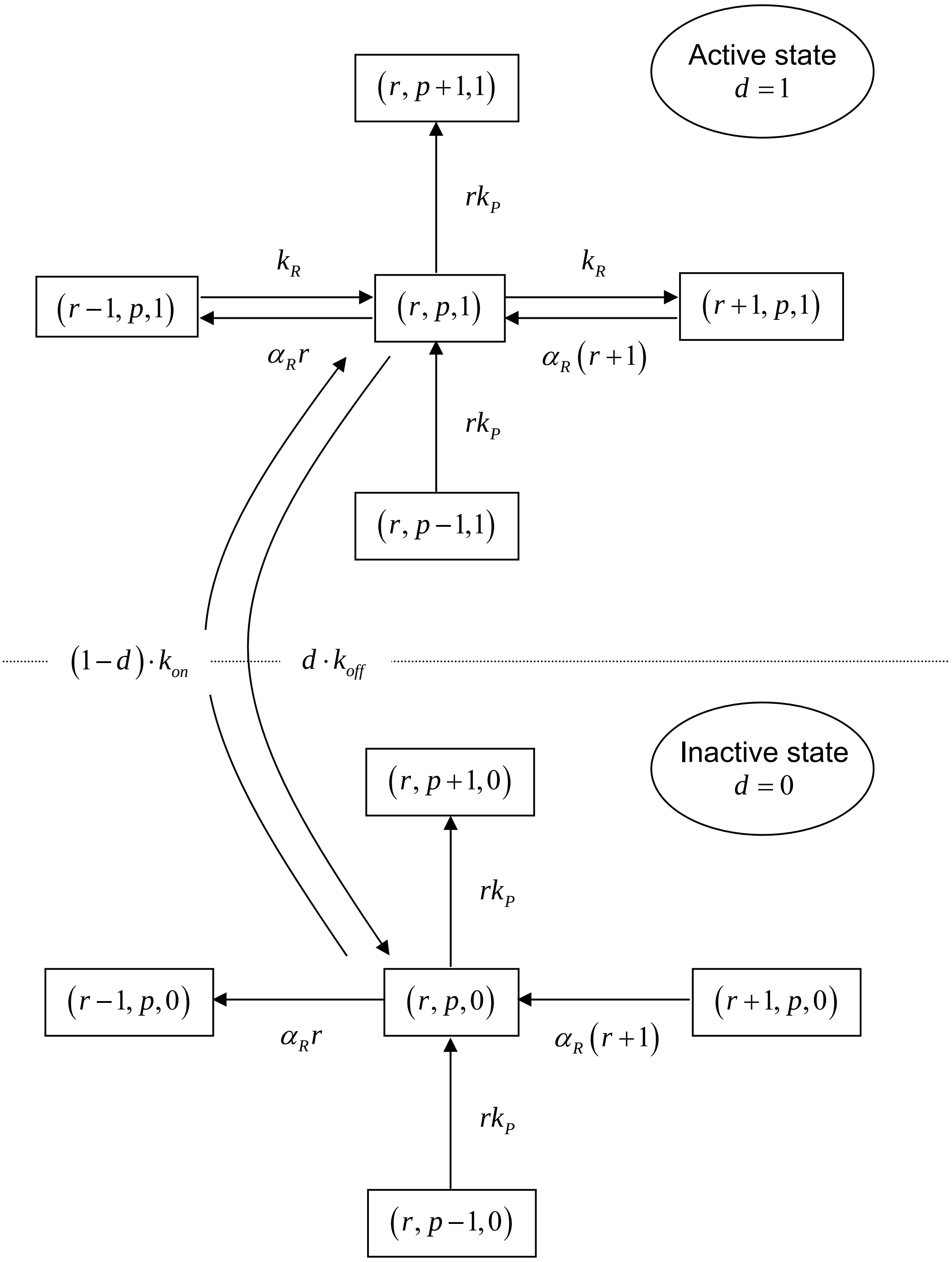

Figure S12: State diagram of the stochastic model including random gene activation-inactivation. The production of mRNA [reactions $(r-1,p,d)\rightarrow(r,p,d)$ and $(r,p,d)\rightarrow(r+1,p,d)$] is possible only when the gene is in active state.

Table S6: The generating function of $f_{r,p,d}$ and all its derivatives (where $F_d(x,y)=\sum_{r,p=0}^{\infty} f_{r,p,d}x^r y^p$ and $d\in\{0,1\}$). The sum of the two generating functions $F(x,y)=F_0(x,y)+F_1(x,y)$ describes the total probability $f_{r,p}=f_{r,p,0}+f_{r,p,1}$ for the number of proteins and mRNA. Note that the generating functions for each state alone are not normalized to 1; rather, substitution of $x=y=1$ gives the probabilities to be in states $d=0$ and $d=1$: $F_0(1,1)=P_0$ and $F_1(1,1)=P_1$.

Generating function	Coefficient of $x^r y^p$	Substitution of $x=y=1$
$F_d(x,y)=\sum_{r,p=0}^{\infty} f_{r,p,d}x^r y^p$	$f_{r,p,d}$	$F_d(1,1)=\sum_{r,p=0}^{\infty} f_{r,p,d}=P_d$<br>$F_0(1,1)+F_1(1,1)=P_0+P_1=1$<br>$\langle d\rangle=0\cdot P_0+1\cdot P_1=P_1$<br>$\langle d^2\rangle=0^2\cdot P_0+1^2\cdot P_1=P_1$
$\dot{F}_d=\sum_{r,p=0}^{\infty}\dot{f}_{r,p,d}x^r y^p$	$\frac{df_{r,p,d}}{dt}$	$\dot{F}_d\Big\vert_{1,1}=\sum_{r,p=0}^{\infty}\dot{f}_{r,p,d}=\frac{d}{dt}\sum_{r,p=0}^{\infty} f_{r,p,d}=\frac{d}{dt}P_d$<br>$\dot{F}_0\Big\vert_{1,1}+\dot{F}_1\Big\vert_{1,1}=\frac{d}{dt}(P_0+P_1)=\frac{d}{dt}(1)=0$
$\frac{\partial F_d}{\partial x}=\sum_{r,p=0}^{\infty} f_{r,p,d}rx^{r-1}y^p$	$(r+1)f_{r+1,p,d}$	$\frac{\partial F_d}{\partial x}\Big\vert_{1,1}=\sum_{r,p=0}^{\infty} f_{r,p,d}r=\langle r\rangle_d$<br>$\frac{\partial F_0}{\partial x}\Big\vert_{1,1}+\frac{\partial F_1}{\partial x}\Big\vert_{1,1}=\langle r\rangle_0+\langle r\rangle_1=\langle r\rangle$
$\frac{\partial \dot{F}_d}{\partial x}=\sum_{r,p=0}^{\infty} \dot{f}_{r,p,d}rx^{r-1}y^p$		$\frac{\partial \dot{F}_d}{\partial x}\Big\vert_{1,1}=\langle \dot{r}\rangle_d$<br>$\frac{\partial \dot{F}_0}{\partial x}\Big\vert_{1,1}+\frac{\partial \dot{F}_1}{\partial x}\Big\vert_{1,1}=\langle \dot{r}\rangle_0+\langle \dot{r}\rangle_1=\langle \dot{r}\rangle$
$x\frac{\partial F_d}{\partial x}=\sum_{r,p=0}^{\infty} f_{r,p,d}rx^r y^p$	$rf_{r,p,d}$	$x\frac{\partial F_d}{\partial x}\Big\vert_{1,1}=\sum_{r,p=0}^{\infty} f_{r,p,d}r=\langle r\rangle_d$<br>$x\frac{\partial F_0}{\partial x}\Big\vert_{1,1}+x\frac{\partial F_1}{\partial x}\Big\vert_{1,1}=\langle r\rangle_0+\langle r\rangle_1=\langle r\rangle$
$xF_d=\sum_{r,p=0}^{\infty} f_{r,p,d}x^{r+1}y^p$	$f_{r-1,p,d}$	$xF_d\Big\vert_{1,1}=\sum_{r,p=0}^{\infty} f_{r,p,d}=P_d$<br>$xF_0\Big\vert_{1,1}+xF_1\Big\vert_{1,1}=P_0+P_1=1$

$xy\frac{\partial F_d}{\partial x} = \sum_{r,p=0}^{\infty} f_{r,p,d}\, r x^r y^{p+1}$	$rf_{r,p-1,d}$	$xy\frac{\partial F_d}{\partial x}\Big\vert_{1,1} = \sum_{r,p=0}^{\infty} f_{r,p,d}\, r = \langle r\rangle_d$ $xy\frac{\partial F_0}{\partial x}\Big\vert_{1,1} + xy\frac{\partial F_1}{\partial x}\Big\vert_{1,1} = \langle r\rangle_0 + \langle r\rangle_1 = \langle r\rangle$
$\frac{\partial^2 F_d}{\partial x^2} = \sum_{r,p=0}^{\infty} f_{r,p,d}\, r(r-1)x^{r-2}y^p$		$\frac{\partial^2 F_d}{\partial x^2}\Big\vert_{1,1} = \sum_{r,p=0}^{\infty} f_{r,p,d}\, r(r-1) = \langle r(r-1)\rangle_d = \langle r^2\rangle_d - \langle r\rangle_d$ $\frac{\partial^2 F_0}{\partial x^2}\Big\vert_{1,1} = \langle r(r-1)\rangle_0 + \langle r(r-1)\rangle_1 = \langle r(r-1)\rangle$
$\left(\frac{\partial^2 \dot{F}_d}{\partial x^2}\right) = \sum_{r,p=0}^{\infty} f_{\dot{r},p,d}\, r(r-1)x^{r-2}y^p$		$\left(\frac{\partial^2 \dot{F}_d}{\partial x^2}\Big\vert_{1,1}\right) = \langle r(r\dot{-}1)\rangle_d = \left(\langle r^2\rangle_d \dot{-} \langle r\rangle_d\right)$ $\frac{\partial^2 \dot{F}_0}{\partial x^2}\Big\vert_{1,1} + \frac{\partial^2 \dot{F}_1}{\partial x^2}\Big\vert_{1,1} = \langle r(r\dot{-}1)\rangle_0 + \langle r(r\dot{-}1)\rangle_1 = \langle r(r\dot{-}1)\rangle$
$\frac{\partial^2 F_d}{\partial x \partial y} = \sum_{r,p=0}^{\infty} rpf_{r,p,d}\, x^{r-1}y^{p-1}$		$\frac{\partial^2 F_d}{\partial x \partial y}\Big\vert_{1,1} = \sum_{r,p=0}^{\infty} rpf_{r,p,d} = \langle rp\rangle_d$ $\frac{\partial^2 F_0}{\partial x \partial y}\Big\vert_{1,1} + \frac{\partial^2 F_1}{\partial x \partial y}\Big\vert_{1,1} = \langle rp\rangle_0 + \langle rp\rangle_1 = \langle rp\rangle$

Table S7: Equations for time-dependent moments $\langle r \rangle$, $\langle p \rangle$, $\langle r^2 \rangle$, and $\langle p^2 \rangle$**.** The moments $\langle r \rangle$, $\langle p \rangle$, $\langle r^2 \rangle$, $\langle rp \rangle$, and $\langle p^2 \rangle$ are given by summing up the averages $\langle ... \rangle_d$ over the two states $d \in \{0,1\}$: $\langle r \rangle = \langle r \rangle_0 + \langle r \rangle_1$, $\langle p \rangle = \langle p \rangle_0 + \langle p \rangle_1$, $\langle r^2 \rangle = \langle r^2 \rangle_0 + \langle r^2 \rangle_1$, $\langle rp \rangle = \langle rp \rangle_0 + \langle rp \rangle_1$, and $\langle p^2 \rangle = \langle p^2 \rangle_0 + \langle p^2 \rangle_1$.

Equation	How to derive this equation from the master equation.
(1) $\dot{P}_1 = k_{on} P_0 - k_{off} P_1$<br>(2) $\dot{P}_0 = k_{off} P_1 - k_{on} P_0$	substitute $x = y = 1$
(3) $\dot{\langle r \rangle}_1 = k_R P_1 - \alpha_R \langle r \rangle_1 + k_{on} \langle r \rangle_0 - k_{off} \langle r \rangle_1$<br>(4) $\dot{\langle r \rangle}_0 = \quad - \alpha_R \langle r \rangle_0 + k_{off} \langle r \rangle_1 - k_{on} \langle r \rangle_0$<br>(3+4) $\dot{\langle r \rangle} = k_R P_1 - \alpha_R \langle r \rangle$	Differentiate the master equation with respect to x, and substitute $x = y = 1$.
(5) $\dot{\langle r(r-1) \rangle}_1 = 2k_R \langle r \rangle_1 - 2\alpha_R \left(\langle r^2 \rangle_1 - \langle r \rangle_1 \right) + ...$<br>$... + k_{on} \langle r(r-1) \rangle_0 - k_{off} \langle r(r-1) \rangle_1$<br>(6) $\dot{\langle r(r-1) \rangle}_0 = \quad -2\alpha_R \left(\langle r^2 \rangle_0 - \langle r \rangle_0 \right) + ...$<br>$... + k_{off} \langle r(r-1) \rangle_1 - k_{on} \langle r(r-1) \rangle_0$<br>(5+6) $\dot{\langle r(r-1) \rangle} = 2k_R \langle r \rangle_1 - 2\alpha_R \left(\langle r^2 \rangle - \langle r \rangle \right)$	Differentiate twice with respect to x, etc.
(7) $\dot{\langle p \rangle}_1 = k_P \langle r \rangle_1 + k_{on} \langle p \rangle_0 - k_{off} \langle p \rangle_1$<br>(8) $\dot{\langle p \rangle}_0 = k_P \langle r \rangle_0 + k_{off} \langle p \rangle_1 - k_{on} \langle p \rangle_0$<br>(7+8) $\dot{\langle p \rangle} = k_P \langle r \rangle$	Differentiate with respect to y
(8) $\dot{\langle p(p-1) \rangle}_1 = 2k_P \langle rp \rangle_1 + k_{on} \langle p(p-1) \rangle_0 - k_{off} \langle p(p-1) \rangle_1$<br>(9) $\dot{\langle p(p-1) \rangle}_0 = 2k_P \langle rp \rangle_0 + k_{off} \langle p(p-1) \rangle_1 - k_{on} \langle p(p-1) \rangle_0$<br>(8+9) $\dot{\langle p(p-1) \rangle} = 2k_P \langle rp \rangle$	Differentiate twice with respect to y.
(10) $\dot{\langle rp \rangle}_1 = k_R \langle p \rangle_1 + k_P \langle r^2 \rangle_1 - \alpha_R \langle rp \rangle_1 + k_{on} \langle rp \rangle_0 - k_{off} \langle rp \rangle_1$<br>(11) $\dot{\langle rp \rangle}_0 = \quad k_P \langle r^2 \rangle_0 - \alpha_R \langle rp \rangle_0 + k_{off} \langle rp \rangle_1 - k_{on} \langle rp \rangle_0$<br>(10+11) $\dot{\langle rp \rangle} = k_R \langle p \rangle_1 + k_P \langle r^2 \rangle - \alpha_R \langle rp \rangle$	Differentiate with respect to x and then y

Analytical solution for time-dependent moments:

Solving the differential equations in Table S8 for YB1 ($\alpha_R > 0$) and PRC1 ($\alpha_R = 0$), we obtain the time-dependent mean and variance of protein and mRNA in the cell population. From these quantities we calculate the Noise strength $\mathrm{NS}(p) = \langle \Delta p^2 \rangle / \langle p \rangle$ and Coefficient of variance $\mathrm{CV}(p) = \langle \Delta p \rangle / \langle p \rangle$ of protein levels, as shown in Table S8. Again, it can be seen that asymptotically (i.e., for $t \gg 1/\alpha_R$), the mean protein level of YB1 is increasing linearly with time ($\langle p \rangle \sim t$), whereas for PRC1 it grows quadratically ($\langle p \rangle \sim t^2$).

As in the previously described model, the non-Poissonian parameter $b \equiv \langle \Delta p^2 \rangle / \langle p \rangle - 1$ is asymptotically constant for YB1, whereas for PRC1 it grows linearly with time. The CV is asymptotically decreasing with time for both cases: $\langle \Delta p \rangle / \langle p \rangle \sim 1/\sqrt{t}$.

Table S8: Mean asymptotic time-dependent levels of protein $\langle p \rangle$, mRNA $\langle r \rangle$, and gene activity $\langle d \rangle$ and their respective noise strengths. Note that the mRNA levels are also non-Poissonian (i.e., Noise strength larger than 1) in this case due to the telegraph process of gene activation and inactivation. Note also that the Noise strength of the telegraph process is smaller than 1. The results of the simple model previously summarized in Table S4 correspond to the case of $P_1 \approx 1$, which occurs when the gene inactivation rate is very small compared to the activation rate: $k_{on} \gg k_{off}$. The meanings of symbols P_1, b_R, and b_P are given in Table S9.

	**YB1**<br>Short mRNA lifetime	**PRC1**<br>Stable mRNA ($\alpha_R \to 0$)
The probability P_d to be in an open/closed configuration.	$P_1 = \frac{k_{on}}{k_{on}+k_{off}}$, $P_0 = \frac{k_{off}}{k_{on}+k_{off}}$	$P_1 = \frac{k_{on}}{k_{on}+k_{off}}$, $P_0 = \frac{k_{off}}{k_{on}+k_{off}}$
Mean gene activity $\langle d \rangle$	P_1	P_1
Mean $\langle r \rangle$	$P_1 \frac{k_R}{\alpha_R}$	$P_1 k_R \cdot t$
Mean $\langle p \rangle$	$P_1 k_P \frac{k_R}{\alpha_R} \cdot t$	$\frac{k_P k_R}{2} P_1 \cdot t^2$
Noise strength $\frac{\langle \Delta d^2 \rangle}{\langle d \rangle}$	$1 - P_1$	$1 - P_1$
Noise strength $\frac{\langle \Delta r^2 \rangle}{\langle r \rangle}$	$1 + \frac{k_{off} k_R}{(k_{off}+k_{on})(k_{off}+k_{on}+\alpha_R)} =$<br>$= 1 + b_R \frac{k_{on}}{k_{on}+\alpha_R P_1}(1-P_1)$	$1 + \frac{2k_{off} k_R}{(k_{off}+k_{on})^2} =$<br>$= 1 + 2(1-P_1) b_R$
Noise strength $\frac{\langle \Delta p^2 \rangle}{\langle p \rangle}$	$1 + \frac{2k_P}{\alpha_R}\left(1 + \frac{k_{off} k_R}{(k_{off}+k_{on})^2}\right) =$<br>$= 1 + \frac{2k_P}{\alpha_R}\left[1 + (1-P_1) b_R\right] =$<br>$= 1 + 2b_P\left[1 + (1-P_1) b_R\right]$	$1 + \frac{2k_P}{3} t \cdot \left(1 + \frac{2k_{off} k_R}{(k_{off}+k_{on})^2}\right) =$<br>$= 1 + \frac{2k_P}{3} t \cdot \frac{\langle \Delta r^2 \rangle}{\langle r \rangle} =$<br>$= 1 + \frac{2k_P}{3} t \cdot \left[1 + 2(1-P_1) b_R\right]$

Table S9: Notations used in Table S8.

Symbol	Biological meaning
$P_1 = \frac{k_{on}}{k_{on}+k_{off}}$, $P_0 = \frac{k_{off}}{k_{on}+k_{off}}$	The asymptotic probability to be active/inactive state.
$b_R = \frac{k_R}{k_{off}+k_{on}} = \frac{k_R / k_{off}}{1+k_{on}/k_{off}}$	The mRNA *burst size*: If $k_{on} \ll k_{off}$ this is the average number of mRNA's produced per single gene activation event.
$b_P = \frac{k_P}{\alpha_R}$	The protein *burst size*: The average number of proteins translated from a single mRNA molecule during its lifetime.

Simulations:

Again we simulate the stochastic model using the Gillespie algorithm with the species r, p, d. The reactions and their respective probabilities are detailed in Table S10.

Table S10: The reactions and their probabilities as implemented by the Gillespie algorithm for simulating PRC1 and YB1 gene expression with gene activation and inactivation. We assume that both proteins are stable ($\alpha_P = 0$). For YB1 the mRNA lifetime is short ($\alpha_R \approx 1\,\mathrm{hr}^{-1}$), whereas for PRC1 mRNA is stable ($\alpha_R = 0$). Note that the probability is a product of the velocity of the reaction and the numbers of ways the molecules of the reactants participating in this reaction can interact. For example, for the reaction "DNA produces one mRNA molecule" ($m=1$) the probability is the product of the velocity of the reaction (k_R) and the active DNA concentration (d).

m	Probability	Reaction implementation
1	$a_1 = dk_R$	$r \to r+1$
2	$a_2 = r\alpha_R$	$r \to r-1$
3	$a_3 = rk_P$	$p \to p+1$
4	$a_4 = p\alpha_P$	$p \to p-1$
5	$a_5 = k_{on}(1-d)$	$d \to d+1$ $(0 \to 1)$
6	$a_6 = dk_{off}$	$d \to d-1$ $(1 \to 0)$

Protein-level distribution:

In order to find the time-dependent distribution of the protein levels $p(t)$ we solved analytically for higher moments (up to moment $n=6$). We find asymptotically (i.e. for times longer than $1/\alpha_R$, $1/k_{on}$, and $1/k_{off}$) that the behavior of moments corresponds to that of the negative binomial and the Gamma distributions, as detailed in Tables S11 and S12. Note that it does not matter if we take into account the activation-inactivation process or not - the Gamma distribution fits the stochastic model for both cases, with the difference being captured by the non-Poissonian parameter b.

Table S11: Behavior of moments for the Poisson distribution, the Negative binomial distribution, the Gamma distribution, and the Log-normal distribution. Each of the first three distributions $P(x)$ is defined by two parameters: the mean $\langle x\rangle$ and the parameter b. In the Negative-binomial distribution b corresponds to the deviation from Poisson, i.e., for $b=0$ the distribution converges back to Poisson. For $b \gg 1$ the Negative binomial and the Gamma distributions are alike.

n	Poisson	Negative Binomial
0	$\langle x\rangle$	$\langle x\rangle$
1	$\frac{\langle x(x-1)\rangle}{\langle x\rangle}=\langle x\rangle$	$\frac{\langle x(x-1)\rangle}{\langle x\rangle}=\langle x\rangle+b$
2	$\frac{\langle x(x-1)(x-2)\rangle}{\langle x(x-1)\rangle}=\langle x\rangle$	$\frac{\langle x(x-1)(x-2)\rangle}{\langle x(x-1)\rangle}=\langle x\rangle+2b$
…		
n	$\frac{\langle x(x-1)\cdot\ldots\cdot(x-n+1)(x-n)\rangle}{\langle x(x-1)\cdot\ldots\cdot(x-n+1)\rangle}=\langle x\rangle$	$\frac{\langle x(x-1)\cdot\ldots\cdot(x-n+1)(x-n)\rangle}{\langle x(x-1)\cdot\ldots\cdot(x-n+1)\rangle}=\langle x\rangle+n\cdot b$
n	Gamma	Log-normal
0	$\langle x\rangle$	$\langle x\rangle$
1	$\frac{\langle x^2\rangle}{\langle x\rangle}=\langle x\rangle+b$	$\frac{\langle x^2\rangle}{\langle x\rangle}=\langle x\rangle\cdot e^{\sigma^2}$
2	$\frac{\langle x^3\rangle}{\langle x^2\rangle}=\langle x\rangle+2b$	$\frac{\langle x^3\rangle}{\langle x^2\rangle}=\langle x\rangle\cdot e^{2\sigma^2}$
…		
n	$\frac{\langle x^{n+1}\rangle}{\langle x^n\rangle}=\langle x\rangle+n\cdot b$	$\frac{\langle x^{n+1}\rangle}{\langle x^n\rangle}=\langle x\rangle\cdot e^{n\sigma^2}$

Table S12: A comparison between the Poisson, Negative binomial, Gamma, and the Log-normal distributions[1].

	Poisson	Negative Binomial
Distribution	$P(x)=e^{-\langle x\rangle}\frac{\langle x\rangle^x}{x!}$	$P(x\mid r,p)=\frac{\Gamma(r+x)}{x!\,\Gamma(r)}p^r(1-p)^x$
Mean $\langle x\rangle$	$\langle x\rangle$	$r\frac{1-p}{p}$
Parameter b	0	$\frac{1-p}{p}$
	Gamma	Log-normal
Distribution	$P(x\mid a,b)=\frac{1}{b^a\Gamma(a)}x^{a-1}e^{-x/b}$	$P(x\mid \mu,\sigma)=\frac{1}{x\sigma\sqrt{2\pi}}e^{-(\ln x-\mu)^2/2\sigma^2}$
Mean $\langle x\rangle$	ab	$e^{\mu+\sigma^2/2}$
Parameter b	b	-

[1] In bacteria the distribution was found to be Negative binomial when the lifetimes of the mRNA were taken to be much shorter than those of the protein $\eta=\alpha_P/\alpha_R\ll 1$ (This is usually the case, as bacterial protein are considered stable and their lifetimes are assumed to be roughly 1 cell cycle). The parameters of the distribution have a simple physical meaning: $b=k_P/\alpha_R$ is the *burst size*, or the number of proteins produced by a single mRNA molecule during its short lifetime, and $a=k_R/\alpha_P$ is the *number of bursts per cell cycle*.

Distribution of onset-times:

Eukaryotic genes are assumed to switch between active (open DNA) and inactive states (closed DNA) at rates k_{on} and k_{off}. Transcription of mRNA occurs only when the cell is in open state. Cells that are initially in closed state produce no protein until they switch to open state for the first time.

The probability that a cell which is initially closed will not open at all until time t_0 (from the beginning of the cell cycle) decays exponentially with time, i.e. $P(t_0) \sim \exp(-k_{on}t_0)$. We define the "onset time" for the expression of a gene to be the duration of time that has elapsed from the initiation of the stochastic process until the expressed protein level has exceeded a certain small threshold. Thus, the onset times t_0 for gene expression in a population of cells are exponentially distributed, with the tail of the distribution decaying as $P_{\text{onset}}(t) \sim \exp(-k_{on}t_0)$. This result is demonstrated in Figure S13.

If the telegraph process is initially in equilibrium, than a fraction $P_1 = k_{on} / (k_{on} + k_{off})$ of the cells are initially in open state, and a fraction $P_0 = k_{off} / (k_{on} + k_{off})$ are in close state. Thus the histogram of onset-times for such a case is composed of a peak at $t_0 = 0$ (whose underlying surface is P_1) followed by an exponentially decaying tail (whose underlying surface is P_0).

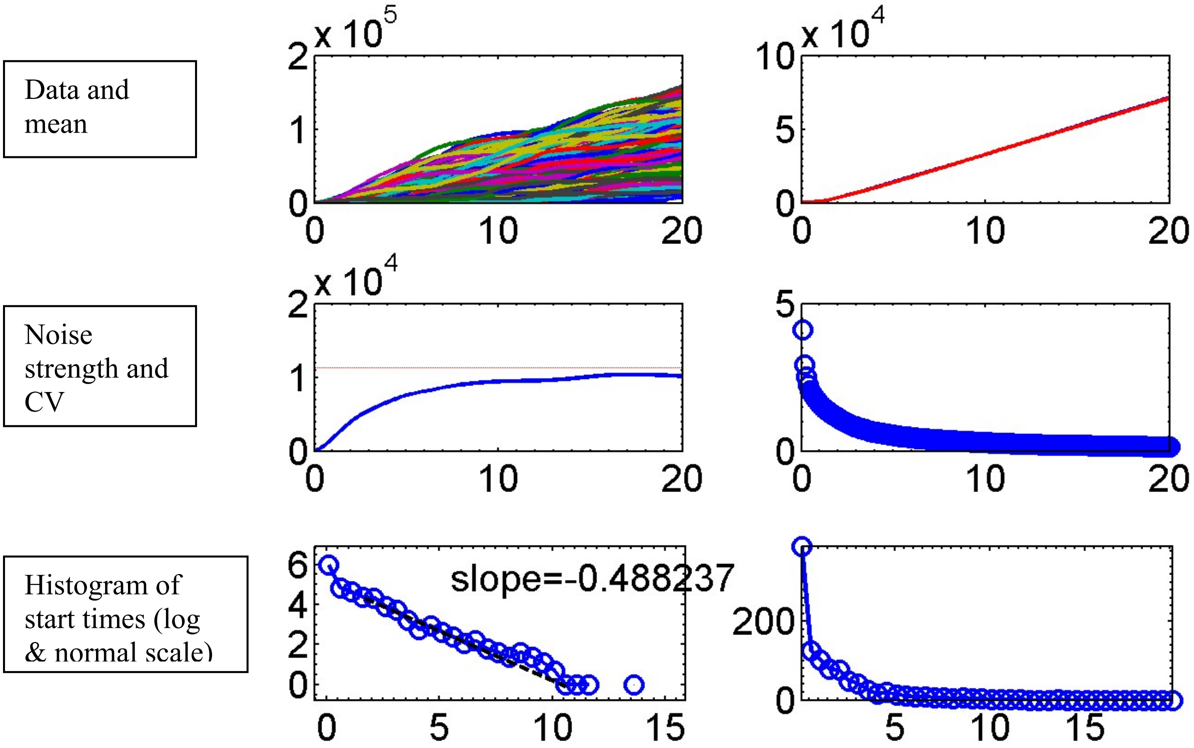

Figure S13: Stochastic simulation for a population of cells expressing a protein with short mRNA lifetime (such as YB1). Simulation parameters are: $k_{off}=1$, $k_{on}=0.5$, $k_R=20$, $\alpha_R=0.7$, $k_P=1$ where the rates are taken in units of events/hour. The onset-times are exponentially distributed as $P_{\text{onset}}(t)\sim\exp(-k_{on}t_0)$. We assume that the telegraph process is in equilibrium throughout the simulation. Note that the exponential decay of the onset times histogram depends only on k_{on} and not on k_{off}. However, if $k_{off}\approx 0$ the surface underneath the exponential tail is $P_0=k_{off}/(k_{on}+k_{off})\approx 0$, and it will disappear. Similar results are achieved for simulations of proteins with stable mRNA (such as PRC1).

We can thus use the histogram of onset times to extract the gene activation rate k_{on} from experimental data. This method is relatively *robust* to changes in the threshold used to calculate the onset time t_0, as demontrated in Figure S14. Furthermore, using the cumulative data function (CDF) gives an estimation that is more robust when the number of cells is small (the PDF is sensitive to empty bins that arise when only 150-200 cells are taken).

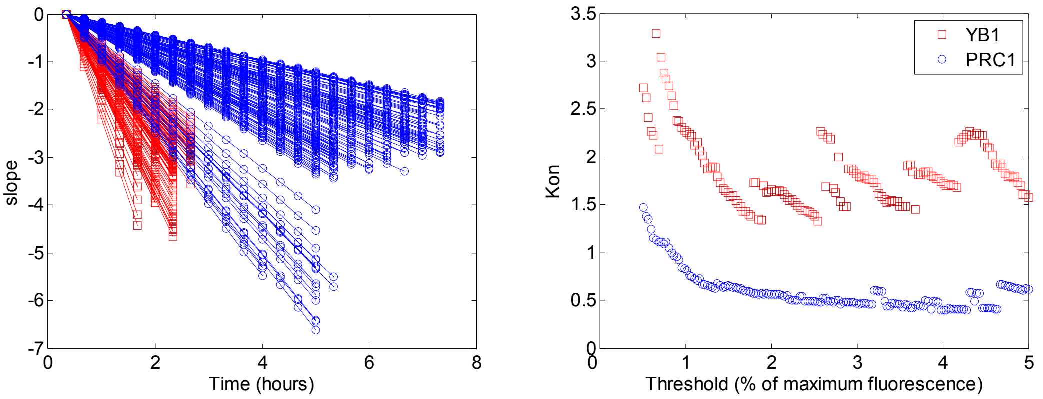

Figure S14: The gene activation rate k_{on} can be robustly extracted from the histogram of onset times t_0 in the above simulation. Shown are extracted values of k_{on} for different thresholds used to calculate t_0. The extracted value of k_{on} is 1.88 ± 0.44 hr^{-1} for YB1 and 0.68 ± 0.24 hr^{-1} for PRC1. For thresholds values up to 5% of the maximal expression level (i.e., the maximal protein level during the entire cell cycle).

Estimation of translation rates:

Assume a gene of length L (in base-pairs) from which a transcript of length L is transcribed. It is known that a single ribosome crawls along the mRNA at a rate[2] of $v = 20\,\text{AA/sec}=60\,\text{bp/sec}$ (Young and Bremer 1976). Thus, a single ribosome translates a new protein every $\tau = \frac{L}{v}$ seconds. We assume that the ribosomes bind to the mRNA at saturating amounts at a varying density[3] of $\rho = 3\cdot 10^{-4} - 3\cdot 10^{-2}\,\text{Ribosomes/bp}$ (Arava, Wang et al. 2003) according to the strength of the ribosome-binding site. Thus, the rate of new proteins being produced is multiplied by the number of ribosomes per transcript: $R = L\rho$. The number of proteins produced per mRNA molecule per second is:

$$k_P = \frac{R}{\tau} = \frac{L\rho}{(L/v)} = v\rho, \qquad \textbf{(S 11)}$$

which gives k_P roughly between 1.8 and 0.018 proteins per second, or $k_P \approx 60 - 6000\,\text{Proteins/hr}$. For PRC1, whose transcript length is roughly 1800bp it has been shown in Yeast to have approximately 10 ribosomes associated on it (http://genome-www.stanford.edu/yeast_translation/). Thus $\rho = 1/180$ Ribosomes/bp and $k_P \approx 1/3\,\text{sec}^{-1} = 1200\,\text{proteins/hr}$.

---

2 This rate, also refered to as the "elongation rate", was measured for bacteria and is assumed to be on the same order of magnitude for yeast and mammalian cells Young, R. and H. Bremer (1976). "Polypeptide-chain-elongation rate in Escherichia coli B/r as a function of growth rate." Biochem J **160**(2): 185-94..

3 Usually ribosome density is taken to be 0.03 to 3 ribosomes per 100 base-pairs. The maximal density corresponds to roughly 30 base-pairs per ribosome. Ribosome density is a roughly decreasing function of the length of the gene Arava, Y., Y. Wang, et al. (2003). "Genome-wide analysis of mRNA translation profiles in Saccharomyces cerevisiae." Proc Natl Acad Sci U S A **100**(7): 3889-94..

Extraction of biochemical parameters - fitting

We have 4 (or 5) biochemical parameters: α_d , k_d , k_R , k_P (and α_R). Note α_p is assumed to be 0.

- We extract k_d from start times histogram.
- We extract α_R from the mean protein level (and independently, from inhibition experiments).
- We are left with: α_d , k_R , k_P

YB1:

We get: $k_d = 1.88$ from the start times histograms.

mRNA lifetime: $\alpha_R \approx 0.75$

Regarding the other parameters, for any value of α_d we get different values of k_R , k_P (and α_R).

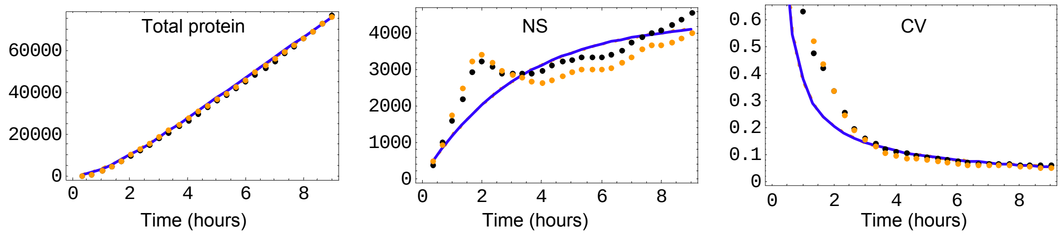

Figure S15: Example of fit (blue line) to total protein, noise strength (NS) and to the coefficient of variance (CV) of experimental data of YB1.

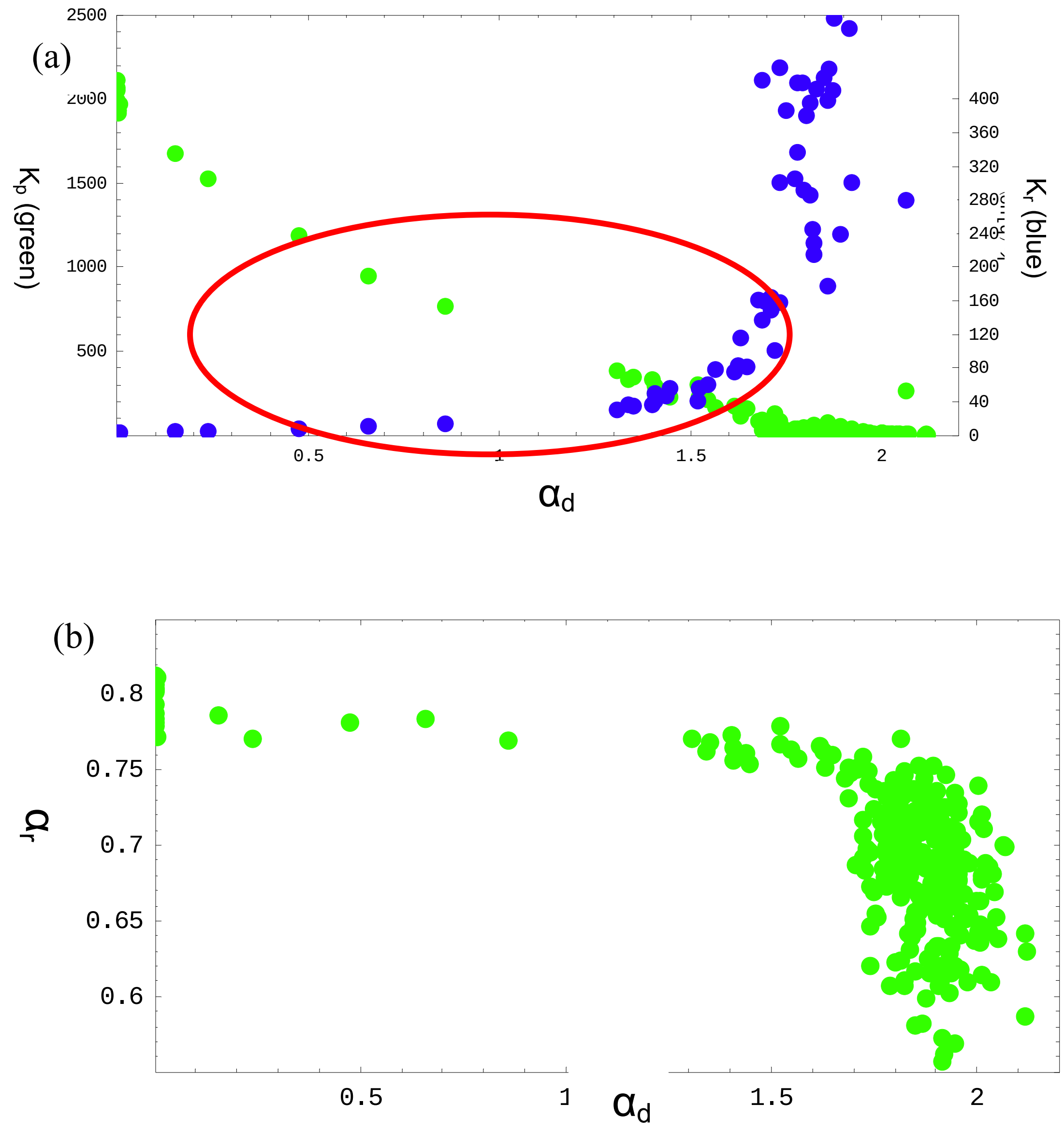

Figure S16: For any value of α_d we get different values of k_R, k_P (a) and α_R (b).

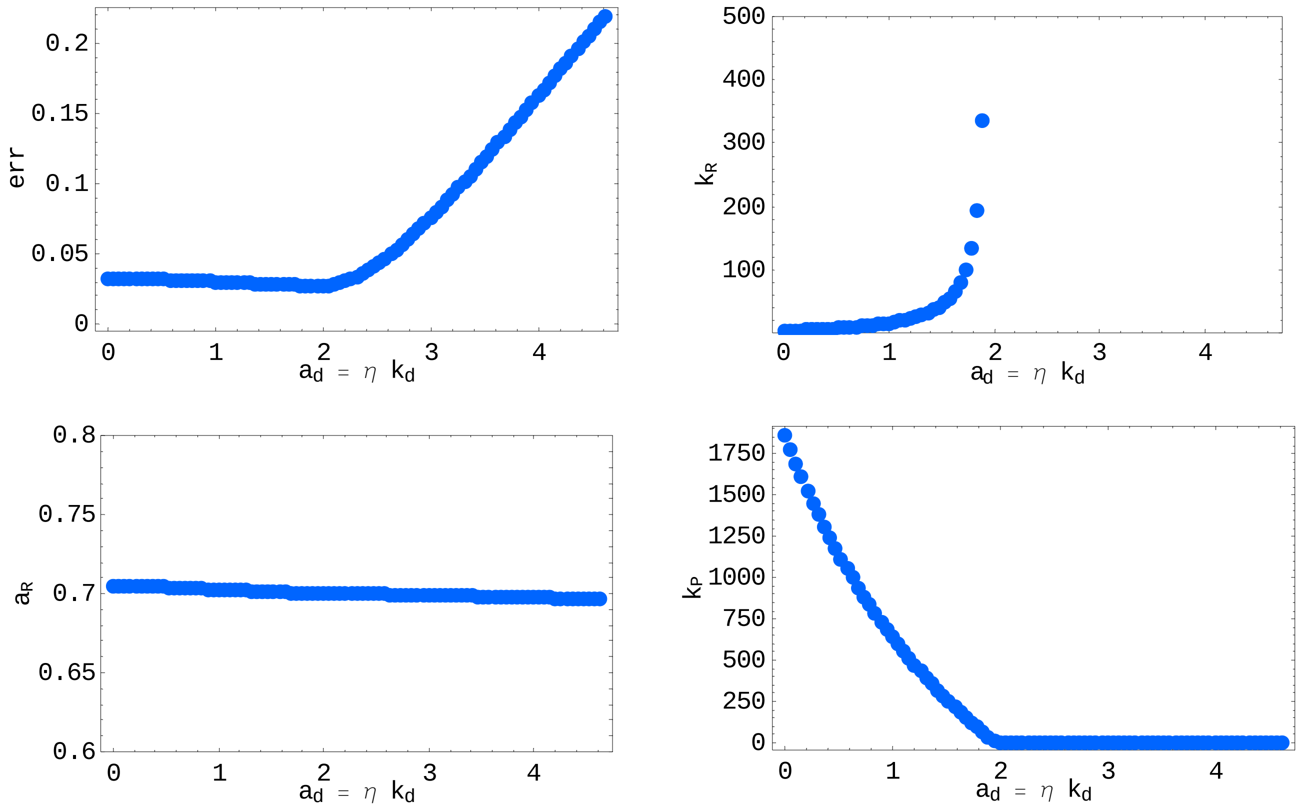

Figure S17: Error "landscape" It can be seen that there is not enough data to specify α_d, k_R, k_P. All values of α_d (in the range $[0, k_d]$) give approximately the same low fit-error to the experimental data. The "potential well" is quite shallow, as can be seen by the bootstrap (i.e. any minima seen inside the well is not robust to addition/deletion of single cells).

PRC1:

We find: $k_d = 0.66$ from the start times histograms.
Regarding the other parameters, for any value of α_d we get different values of k_R, k_P (and α_R).

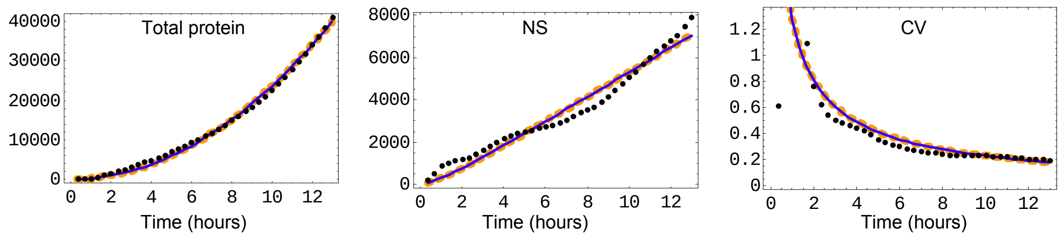

Figure S18: Example of fit (blue line) to total protein, noise strength (NS) and to the coefficient of variance (CV) of experimental data of YB1.

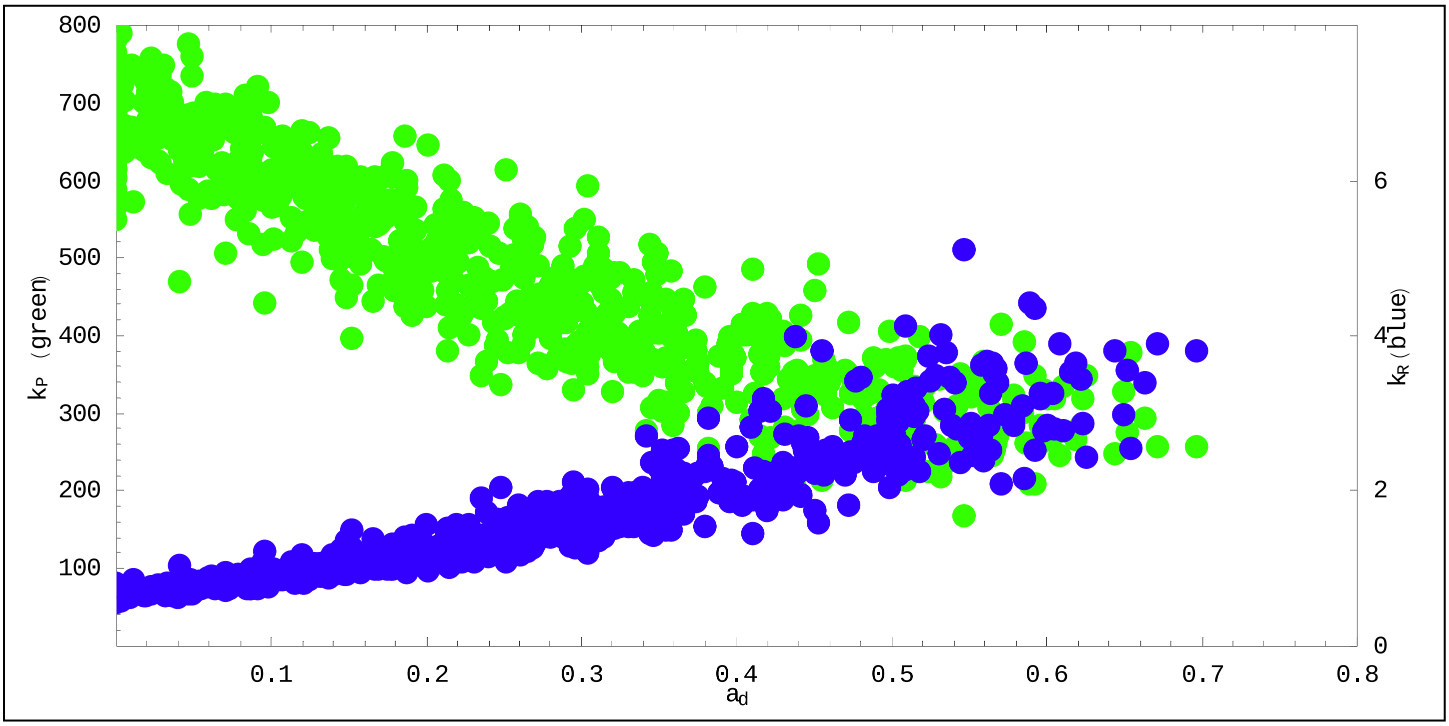

Figure S19: For any value of α_d we get different values of k_R, k_P.

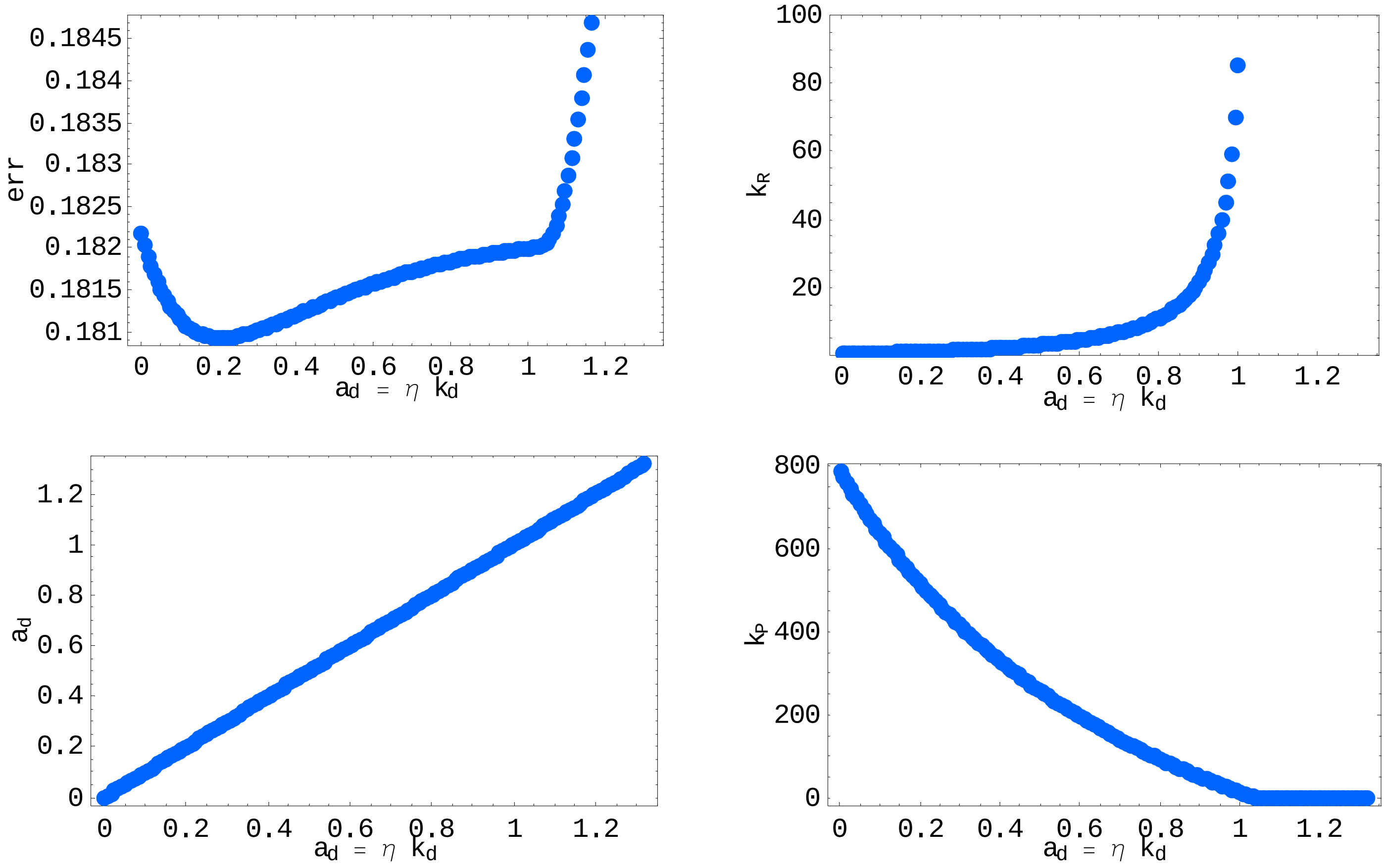

Figure S20: Error "landscape" It can be seen that there is not enough data to specify α_d, k_R, k_P. All values of α_d (in the range $[0, k_d]$) give approximately the same low fit-error to the experimental data. The "potential well" is quite shallow, as can be seen by the bootstrap (i.e. any minima seen inside the well is not robust to addition/deletion of single cells).

However, all values of k_R, k_P inside the "potential well" are biochemically feasible.

Cell-to-cell variability can stem from either extrinsic or intrinsic sources

In order to model the cell-to-cell variability two different models may be suggested. The first model assumes that the observed variability has 'extrinsic' origins, meaning that each cell has different rate constants which are conserved for a relatively long time period. Such differences were suggested to rise for example from amounts of transcriptional and translational machinery that differ between cells (Elowitz, Levine et al. 2002; Pedraza and van Oudenaarden 2005). The second model assumes that the observed variability arises from stochastic processes due to low copy molecular interactions and has been termed 'intrinsic'.

In order to try and characterize the sources of variability in protein accumulation of PRC1 and YB1, we ran intrinsic and extrinsic simulations of the process. Intrinsic simulations were run using the Gillespie algorithm (Gillespie 1977) taking into account DNA opening rate - computed from onset times, and protein and mRNA half lives – computed from the transcription inhibition experiment (as previously described in the supplementary text). Extrinsic simulations were run by fitting linear and quadratic curves to each single cell profile of YB1 and PRC1 respectively, and adding the fit-to-experiment difference as experimental noise.

We find that all measured parameters of the experiment on PRC1 and YB1, including, mean, noise strength, auto correlation and the ergodicity metric all can be reproduced (within the experimental error limit) using feasible biochemical parameters in both extrinsic and intrinsic simulations (see Figures S21-S23).

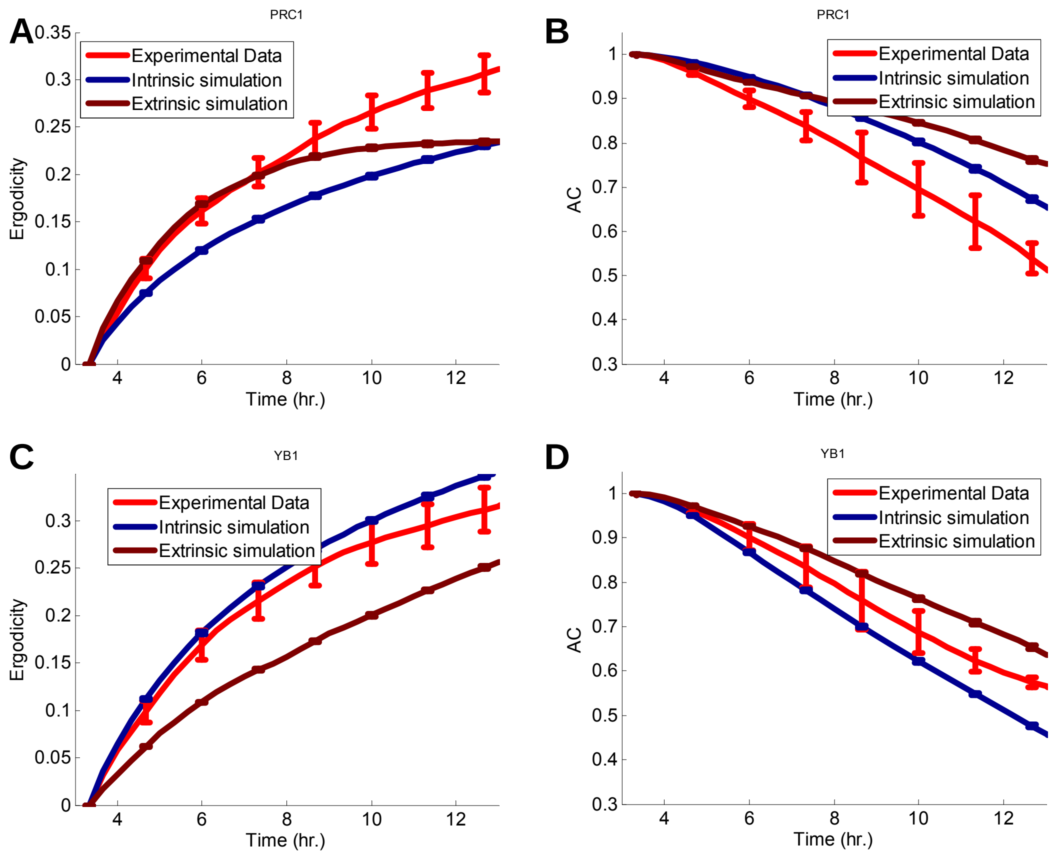

Figure S21: Mixing of cells as observed from experimental data vs. mixing as result of extrinsic or intrinsic sources of cell-to-cell differences. Mixing occurs when a cell lineage, given enough time, reaches the different states found in a snapshot of a cell population. Mixing was measured using two approaches (detailed by Sigal et al. (Sigal, Milo et al. 2006)). The first is the auto-correlation function A(τ) of the protein levels (PRC1 at plot B and YB1 at plot D). The second, an 'ergodicity' metric, ranked the cells according to tagged protein fluorescence at the beginning of the movie, and followed the fraction of the total ranks that each cell traversed as a function of time (PRC1 at plot A and YB1 at plot C). All graphs (A-D) denote measurements of mixing between 3-13 hours following beginning of protein accumulation for PRC1 (A,B) and YB1 (C,D). In each graph, red line denotes average mixing measured in the experiment, brown line denotes mixing measured in simulation with extrinsic origins of noise and blue line denotes mixing measured in intrinsic simulation of a 3 stage molecular process as discussed in the text.

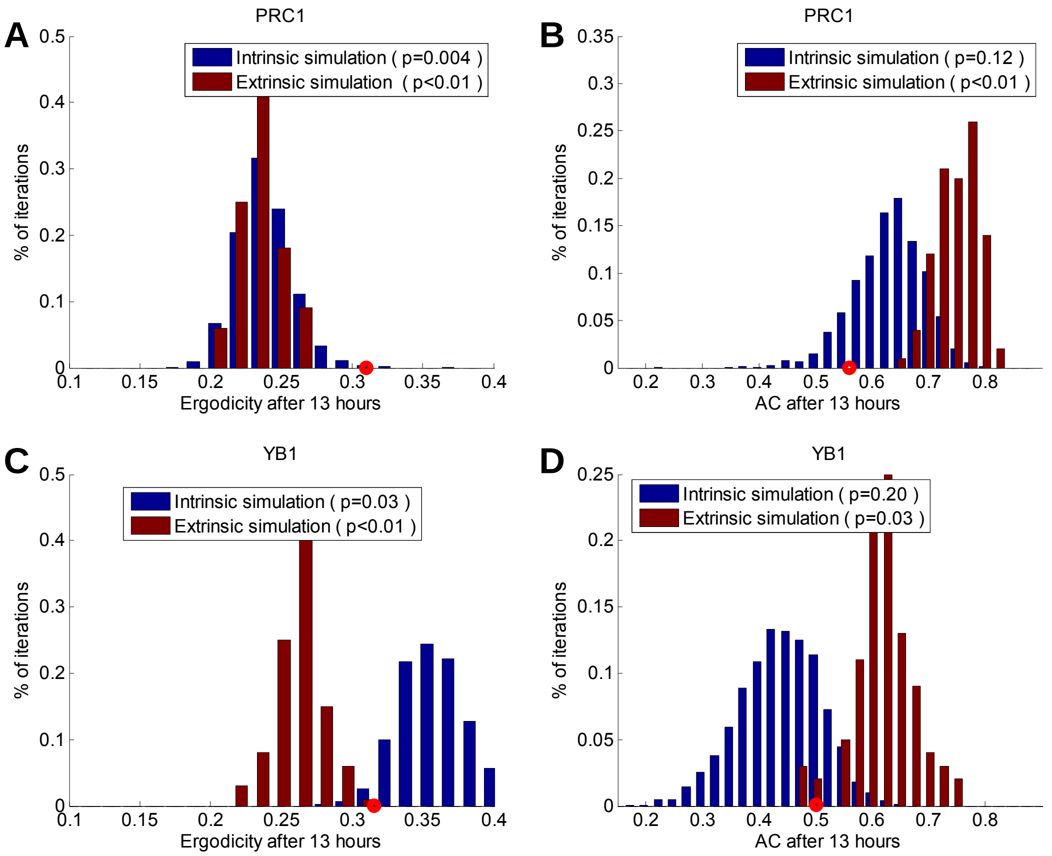

Figure S22: Mixing of cells as observed from experimental data vs. mixing as result of extrinsic or intrinsic sources of cell-to-cell differences. Mixing is measured as detailed in figure S20. All graphs (A-D) denote distributions of mixing after 13 hours of protein accumulation. Mixing measured from extrinsic simulations is brown and from intrinsic simulations is blue. Red circle denotes experimental data A,C) 'Ergodicity' metric measured for PRC1 and YB1 respectively. B,D) Auto correlation measured for PRC1 and YB1 respectively.

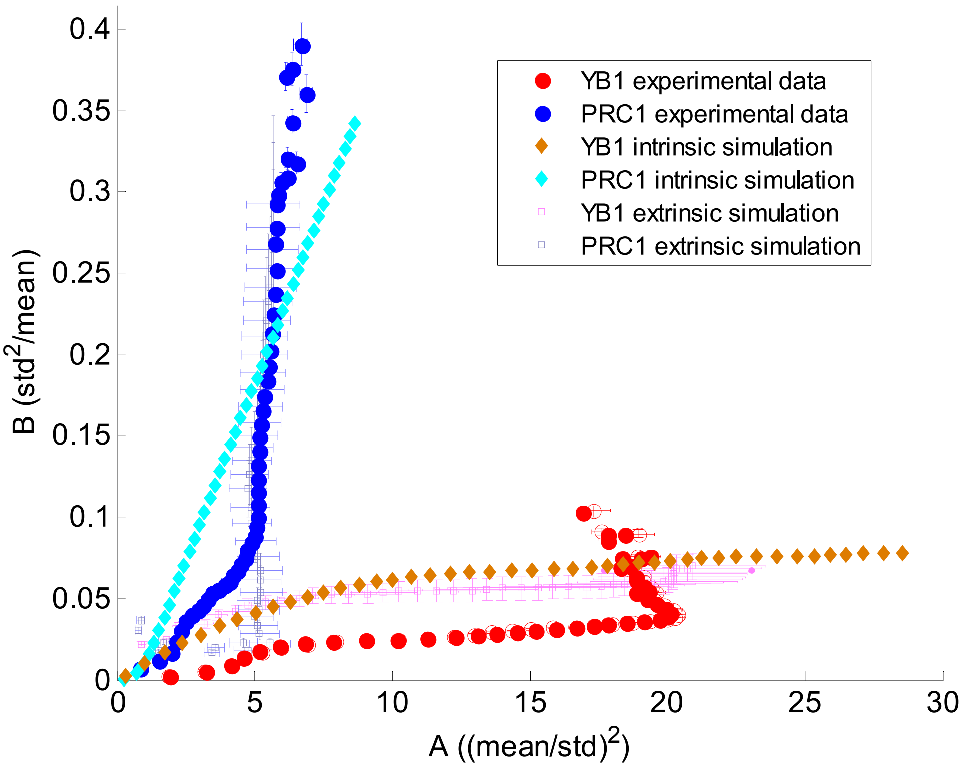

Figure S23: Gamma distribution consists of a scale parameter *a,* and shape parameter *b,* which are both related to the mean, μ, and standard deviation, σ, by $\alpha=\mu^2/\sigma^2$ and $b=\sigma^2/\mu$. Bold blue and red circles denote *a*,*b* measurements for YB1 and PRC1 respectively for the first 12 hours of protein accumulation. Cyan and orange diamonds denote *a*,*b* measurements from intrinsic simulations that best fit the data. Gray and pink squares denote *a*,*b* measurements from extrinsic simulations based on parameters fitted from the experimental data. Error bars denote standard errors.

Supporting movies

Supporting Movie 1:
Time-lapse movie of transmitted light images of the clone with YFP CD-tagged PRC1. Movie duration is 92 hours, each frame is played for 0.1 second (time-lapse: 1 frame per 20 minutes).

Supporting Movie 2:
Time-lapse movie of yellow fluorescence images of the clone with YFP CD-tagged PRC1. Movie duration is 92 hours, each frame is played for 0.1 second (time-lapse: 1 frame per 20 minutes).

Supporting Movie 3:
Time-lapse movie of yellow fluorescence images overlaid on transmitted light images of the clone with YFP CD-tagged PRC1. Movie duration is 92 hours, each frame is played for 0.1 second (time-lapse: 1 frame per 20 minutes).

Supporting Movie 4:
Time-lapse movie of transmitted light images of the clone with YFP CD-tagged YB1. Movie duration is 92 hours, each frame is played for 0.1 second (time-lapse: 1 frame per 20 minutes).

Supporting Movie 5:
Time-lapse movie of yellow fluorescence images of the clone with YFP CD-tagged YB1. Movie duration is 92 hours, each frame is played for 0.1 second (time-lapse: 1 frame per 20 minutes).

Supporting Movie 6:
Time-lapse movie of yellow fluorescence images overlaid on transmitted light images of the clone with YFP CD-tagged YB1. Movie duration is 92 hours, each frame is played for 0.1 second (time-lapse: 1 frame per 20 minutes).

Supporting Movie 7:
Time-lapse movie of transmitted light images of the clone with YFP CD-tagged ANLN. Movie duration is 92 hours, each frame is played for 0.1 second (time-lapse: 1 frame per 20 minutes).

Supporting Movie 8:
Time-lapse movie of yellow fluorescence images of the clone with YFP CD-tagged ANLN. Movie duration is 92 hours, each frame is played for 0.1 second (time-lapse: 1 frame per 20 minutes).

Supporting Movie 9:
Time-lapse movie of yellow fluorescence images overlaid on transmitted light images of the clone with YFP CD-tagged ANLN. Movie duration is 92 hours, each frame is played for 0.1 second (time-lapse: 1 frame per 20 minutes).

Supporting references

Arava, Y., Y. Wang, et al. (2003). "Genome-wide analysis of mRNA translation profiles in Saccharomyces cerevisiae." Proc Natl Acad Sci U S A **100**(7): 3889-94.

Elowitz, M. B., A. J. Levine, et al. (2002). "Stochastic gene expression in a single cell." Science **297**(5584): 1183-6.

Gillespie, D. E. s. s. o. c. c. r. (1977). **81**: 2340.

Nguyen, V. T., F. Giannoni, et al. (1996). "In vivo degradation of RNA polymerase II largest subunit triggered by alpha-amanitin." Nucleic Acids Res **24**(15): 2924-9.

Peccoud, J. and B. Ycart (1995). "Markovian modeling of gene-product synthesis." **48**: 222-234.

Pedraza, J. M. and A. van Oudenaarden (2005). "Noise propagation in gene networks." Science **307**(5717): 1965-9.

Young, R. and H. Bremer (1976). "Polypeptide-chain-elongation rate in Escherichia coli B/r as a function of growth rate." Biochem J **160**(2): 185-94.
